# Supplementary material for: Red blood cell distribution width as a risk factor for inhospital mortality in obstetric patients admitted to an intensive care unit: a single centre retrospective cohort study
Source: BMJ Open. 2017 Jun 21;7(6):e012849. doi: 10.1136/bmjopen-2016-012849 (PMC5577870; doi:10.1136/bmjopen-2016-012849)
Supplement: Author's manuscript [file bmjopen-2016-012849.draft_revisions.pdf]

BMJ Open

Red cell distribution width improves prognostic performance of the Acute Physiology and Chronic Health Evaluation II score in obstetric patients admitted to intensive care unit

Journal:	*BMJ Open*
Manuscript ID	bmjopen-2016-012849
Article Type:	Research
Date Submitted by the Author:	27-May-2016
Complete List of Authors:	Chu, Yufeng; Shandong Provincial Hospital Affiliated to Shandong University<br>Yuan, Zhongshang; Shandong University, Department of Epidemiology and Biostatistics<br>Meng, Mei; Shandong Provincial Hospital Affiliated to Shandong University<br>Zhou, Haiyan; Shandong Tumor Hospital and Institute<br>Wang, Chunting; Shandong Provincial Hospital Affiliated to Shandong University<br>Ren, Hongsheng; Shandong Provincial Hospital Affiliated to Shandong University, Department of Intensive Care Unit
**Primary Subject Heading**:	Intensive care
Secondary Subject Heading:	Obstetrics and gynaecology
Keywords:	APACHE-II score, Critical care, Mortality, Obstetrics, Red cell distribution width

SCHOLARONE™
Manuscripts

For peer review only

TITLE PAGE

Red cell distribution width improves prognostic performance of the Acute Physiology and Chronic Health Evaluation II score in obstetric patients admitted to intensive care unit

Yufeng Chu, [a, *] Zhongshang Yuan, [b, *] Mei Meng, [a] Haiyan Zhou, [c] Chunting Wang,[a] Hongsheng Ren [a]

[a] *Department of Intensive Care Unit, Shandong Provincial Hospital Affiliated to Shandong University, Shandong University, Jinan, P.R. China*

[b] *Department of Epidemiology and Biostatistics, School of Public Health, Shandong University, Jinan, P.R. China*

[c] *Department of Medical Oncology ,Shandong Tumor Hospital, Jinan, P.R. China*

Yufeng Chu and Zhongshang Yuan are co-first authors.

Corresponding author:

Hongsheng Ren, MD, Ph. D.

Department of Intensive Care Unit

Shandong Provincial Hospital Affiliated to Shandong University

No. 324 Jingwu Road, Jinan, Shandong, 250012, P.R. China

Email: sdslicu@163.com

For peer review only

Abstract

Objectives: Red blood cell distribution width (RDW) has been shown to predict mortality in critically ill patients. The primary aim of this study was to examine the association between RDW and ICU mortality in obstetric critical care patients. The secondary aim was to investigate whether adding RDW to Acute Physiology and Chronic Health Evaluation II (APACHE II) improves the prognostic performance of APACHE II.

Setting: Single center, retrospective observational study in China.

Participants: A total of 447 consecutive obstetric patients admitted in the ICU from January 1, 2008 to December 31, 2013 were included. Patients were excluded if they had known hematologic diseases, history of recent blood transfusion, and patients who died or were discharged from ICU within 24 hours of admission.

Primary outcome measures: Each patient's ICU entrance characteristics including RDW were retrieved. APACHE II scores were calculated using the worst value of 12 acute physiological variables within 24 hours of presentation. The primary end point of the study was ICU mortality. The primary predictor of interest was RDW and APACHE II scores at ICU admission.

Results: A total of 376 patients were included in this present study. ICU mortality was 5.32%. A significant association was found between baseline RDW levels and ICU mortality (hazard ratio per percent increase in RDW, 1.21; 95% CI, 1.02 to 1.57). After categorization based on tertile of baseline RDW, as well as further adjustment for hematocrit and other risk factors, a

For peer review only

graded independent association between RDW and mortality was observed (*P*<0.001). The addition of RDW to the APACHE II score improved the AUC for ICU mortality from 0.766 to 0.872 (*P*<0.001).

Conclusions: RDW is an independent predictor for ICU mortality in Chinese obstetric critical care patients. Combining RDW to APACHE II scores could further improve its prognostic performance.

Key words: APACHE-II score; Critical care; Mortality; Obstetrics; Red cell distribution width

Strengths and limitations of this study

• Red cell distribution width (RDW), a quantitative measure of erythrocyte size variability, is an independent prognostic outcome predictor in obstetric critical care patients.

• RDW significantly improved the prognostic accuracy of the APACHE II score in obstetric critical care patients.

• The study was a single-center study. It was very heterogenous, and patient numbers were relatively small, so large epidemiologic study may be required to validate the findings.

For peer review only

Introduction

Despite the advances in the care of obstetric patients, maternal mortality ratio (MMR) has remained high, especially in developing countries.[1, 2] The use of scoring systems to assess severity and predict mortality may help identify obstetric patients who truly require intensive care.[3] Acute Physiology and Chronic Health Evaluation II (APACHE II) scores are predictive scores for mortality that are widely used in the general population. However, results from obstetric patients have shown some inconsistencies; wherein, some studies have shown that these scores are a good predictor of illness severity, while more studies have shown that these scores overestimated mortality.[4, 5, 6, 7-9] Further work is need to improve the prognostic accuracy of APACHE II scores in obstetric patients.

Red cell distribution width (RDW) is a parameter that is easy and inexpensive to obtain, and reflects the degree of heterogeneity of erythrocyte volume.[10] RDW was used in the differential diagnosis of anemia in the past decades.[11] Recently, RDW has been shown to be a novel, independent prognostic marker for mortality, mainly in patients with cardiovascular disease and strokes, as well as in critically ill patients.[12-19] However, few ICU subpopulations have been studied so far. The obstetric population is of special interest because of the limitations of existing scoring system, which largely are based on physiological parameters.

Therefore, we conducted a retrospective study to evaluate the prognostic value of RDW, and investigated whether adding RDW could improve the prognostic performance of APACHE-II scores in critically ill obstetric patients.

For peer review only

Methods

This study was approved by the Institutional Review Board of the Provincial Hospital Affiliated to Shandong University. Obstetric patients consecutively admitted in the ICU for at least 24 hours from January 1, 2008 to December 31, 2013 were included in this retrospective observational study. The requirement for patient consent was waived because this study did not affect the patient's clinical care, and all protected health information was deleted. Obstetric patients were defined as pregnant women or up to six weeks postpartum. This study carried out at the Shandong Provincial Hospital Affiliated to Shandong University, Jinan, China. The hospital is a 1500-bed tertiary academic hospital with 20 ICU beds. The hospital provides primary as well as tertiary care to an ethnically and socioeconomically diverse population within Shandong province and the surrounding region. The decision to transfer patients into the ICU was made by at least one senior critical care doctor and one senior obstetric doctor. Likewise, these doctors also made decisions to discharge patients or to transfer patients to general wards. Patients were excluded if they had known hematologic diseases (including leukemia, thrombotic thrombocytopenic purpura, and other hematologic diseases) or history of recent blood transfusion (less than two weeks). Patients who died or were discharged from the ICU within 24 hours of admission were excluded, because data was difficult to obtain from these patients.

Data collection

For peer review only

Demographic and clinical characteristics including age, gestational age, parity, pregnancy status at admission, diagnosis at entry and during ICU stay, and length of hospital stay were collected. The cesarean section was considered an emergency intervention. Diagnosis of acute kidney injury (AKI) was based on the Acute Kidney Injury Network (AKIN) criteria (20). AKI was defined as an absolute increase in serum creatinine of more than or equal to 0.3 mg/dl, a percentage increase in serum creatinine of more than or equal to 50% (1.5 fold from baseline) within 48 hours, or reduction in urine output (documented oliguria of less than 0.5 ml/Kg per hour for more than six hours).[20] APACHE II scores were calculated using the worst value of 12 acute physiological variables within 24 hours of presentation. These variables included temperature, blood pressure, heart rate, respiratory rate, arterial oxygenation, arterial pH, serum sodium, serum potassium, serum creatinine, hematocrit, white blood cell count, and Glasgow Coma scores. If the patient was sedated at the time of ICU admission, the last Glasgow Coma Scale obtained prior to sedation was collected. RDW, hemoglobin level, hematocrit and mean corpuscular volume (MCV) of all patients included in this study were determined at admission using a Beckman Coulter LH-750 Hematology Analyzer (Beckman Coulter, Inc., Fullerton, California), as a part of the complete blood cell count. Normal reference range for RDW in the hospital laboratory is 10.9% to 15.4%.

Study outcomes

All patients were followed up during hospitalization. The primary end point of the study was ICU mortality. The primary predictor of interest was RDW and APACHE II scores at ICU admission.

For peer review only

Statistics

All continuous variables were presented as means ± standard deviations, or medians with interquartile ranges as appropriate. Categorical data were summarized as number or percentage. We divided RDW into tertiles, and compared the demographics, diagnosis, clinical characteristics, laboratory test results, and APACHE-II scores of patients using analysis of variance or Kruskal-Wallis tests for continuous variables, and Chi-square or Fisher's exact tests for categorical variables. Univariate logistic regression analysis was conducted to examine the association between mortality and each of the predictors, separately. Multivariate logistic regression was further utilized to determine independent predictors of ICU mortality after adjusting for potential confounding factors. An appealing receiver operating characteristic (ROC) curve was used to examine the performance of APACHE-II scores and RDW in predicting ICU mortality. The curve represented a sensitivity plot *versus* 1-specificity. The area under the curve (AUC) was derived from the ROC curve, and the Youden index was adopted to define the optimal cut-off value.[21] We also constructed an ROC curve for the combined APACHE-II score and RDW results for predicting ICU mortality according to the weighted sum formula derived from multivariate logistic regression: logit (mortality) = 0.162 x APACHE-II score + 0.203 x RDW - 7.503; wherein, logit (mortality) is the logarithm of the odds of a critically ill obstetric patient dying in the ICU. Differences between the AUC were detected by Delong's test.[22] Two-sided *P*-values <0.05 were considered statistically significant, and all analyses were performed with R software (http://www.cran.r-project.org/).

For peer review only

Results

Population

A total of 447 obstetric patients who were admitted to ICU were initially enrolled in this study. ICU admission rate was 21.73 per 1,000 births. Eight patients who were diagnosed with hematologic disease and 59 patients who received red blood cell transfusion within two weeks were excluded. Two patients who died within 24 hours after ICU admission were also excluded. Two patients were excluded for missing data. In the final analysis, 376 patients were included in this study.

Association between RDW and ICU mortality

A total of 20 deaths occurred in this cohort during the study period. ICU mortality was 5.32%, and RDW ranged from 10.7% to 24.0% (median, 14.8%; interquartile range, 13.4% to 16.0%).

Participants were divided into three categories on the basis of their baseline tertiles of RDW level: tertile I, 10.7%-13.9%; tertile II, 13.9%-15.6%; tertile III, 15.6%-20.4% (all P<0.001). There was no difference in age, gestational weeks, primary reasons for ICU admission, AKI morbidity, as well as in the total length of stay in hospital (TLSH) among the three tertiles. Hb, MCV and HCT significantly decreased with the increase of RDW, while APACHE II scores and ICU mortality significantly increased with the increase of RDW (Table 1). A significant association was found between baseline RDW levels and ICU mortality (hazard ratio per percent increase in RDW, 1.21; 95% CI, 1.02 to 1.57).

For peer review only

Regression analysis for ICU mortality

Univariate logistic regression analysis demonstrated that patients with higher RDW, higher APACHE-II scores, higher AKI morbidity, and longer TLSH days had significantly greater death hazards (Table 2). Multivariate logistic regression analysis revealed that RDW, AKI and APACHE-II scores were independent predictors of ICU mortality (Table 3). The association of RDW and ICU mortality remained significant after adjusting for age, hemoglobin, MCV, hematocrit, APACHE-II score and AKI. RDW was a significant outcome predictor, which is independent of APACHE-II scores.

A ROC curve was drawn to evaluate the value for RDW and APACHE-II scores in predicting mortality (Figure 1). The AUC was calculated as 0.766 (95% CI, 0.705 to 0.826) for the APACHE-II score and 0.752 (95% CI, 0.684 to 0.862) for RDW. Optimal cut-off value of the APACHE-II score for predicting mortality was five points, which gave a sensitivity of 90.9% and a specificity of 60.7%. The optimal cut-off value of RDW was 16.1%, which gave a sensitivity of 68.2% and specificity of 77.9%. To further clarify whether RDW had an additive power with the APACHE-II score for ICU mortality, we combined RDW and the APACHE-II score to draw a third ROC curve, as shown in Figure 1. Compared with the APACHE-II score, adding RDW to APACHE-II scores improved the AUC from 0.766 to 0.872 (P<0.001). These results suggest that combining RDW and APACHE-II added to the ability to discriminate mortality risk.

Discussion

For peer review only

The main finding of our study was that RDW was independently associated with ICU mortality in obstetric critical care patients of China. This association remained significant even after adjusting for APACHE-II scores, hemoglobin levels, hematocrit and mean corpuscular volume. Combining RDW and the APACHE-II score improved the prognostic accuracy of ICU mortality predicted by APACHE-II alone in the study population.

We found that RDW was independently associated with ICU mortality in a Chinese obstetric ICU population. Patients were excluded if they had a history of recent blood transfusions because RDW could be increased in anemia or after blood transfusions.[11, 23] In this present study, higher levels of RDW were associated with ICU mortality even after adjusting for hemoglobin and hematocrit. In addition, the independent association between RDW and ICU mortality was not eliminated even after adjusting for other potential confounders, including mean corpuscular volume, hemoglobin, hematocrit, APACHE-II score, gestational age and AKI. Our results were consistent with previous analyses of two large populations of critically ill patients.[24, 25] Further, our results were also similar to the findings obtained in a single-center observational study in 2011; [16] wherein, the independent association between RDW and all-cause mortality in critically ill patients was shown.

The important finding in our analysis was that combining RDW and APACHE-II score performed better than APACHE-II score alone in predicting ICU mortality in critically ill obstetric patients. The APACHE-II scoring system developed in 1985 has shown a positive correlation with hospital mortality, and was one of the most common models used for evaluating the severity of a disease in critically ill patients.[26] Previous studies[27-29] suggested APACHE II

For peer review only

score was a good discriminator of illness severity in obstetric cohorts. The sensitivity and specificity of the APACHE II score was evaluated with the use of receiver operating characteristic curve analysis. In accordance with previous studies[27-29], the APACHE-II score was also demonstrated to have a strong power to predict ICU mortality (AUC=0.766) in the present study. Despite its moderate discriminative power alone (AUC=0.752), adding RDW to the APACHE-II score resulted to a better increase in the AUC from 0.766 to 0.872 (P<0.001). Combining RDW with APACHE-II score significantly improved prognostic performance in critically ill obstetric patients. Similarly, another study by Wang *et al.* [16] also revealed that adding RDW to APACHE-II score significantly improves prognostic reliability of APACHE-II score in identifying critically ill patients. Since RDW is a simple, inexpensive, and widely available test as part of the complete blood count, these data may have significant clinical implications for determining prognosis in critically ill obstetric patients.

The pathophysiologic mechanism underlying the association of higher RDW with worse outcomes in critically ill obstetric patients remains unclear. Generally, the increase in RDW reflects either impaired erythropoiesis, abnormal red blood cell survival, or both. Metabolic abnormalities such as shortening of telomere length, poor nutritional status, [30, 31] inflammation, [32-34] oxidative stress, [35, 36] dyslipidemia, hypertension, erythrocyte fragmentation, and alteration of erythropoietin function might contribute to RDW increase.[37] In normal parturition, the increase of RDW might be related to stimulus-induced reticulocytosis in the last few weeks.[29] This might not be the case in our study;

For peer review only

wherein, even if five patients with spontaneous onset of labor were excluded, RDW remained as a significant predictor of mortality.

In this present study, the admission rate of 21.73 per 1,000 births was relatively higher than those reported by other studies, [38-39] and the mean APACHE-II score was relatively lower compared with other groups.[7, 16, 40] The lack of a high dependency unit as a referral center in local areas for complicated pregnancies could partially explain these difference.

This study had several limitations. First, we did not evaluate the evolution of RDW, and thus, it remained unclear whether changes in RDW over time may provide additional prognostic information. Second, we only used the APACHE-II score to predict mortality in obstetric patients, which was limited by spontaneous improvement after delivery in peripartum women, [41-47] and inconsistent results.[48, 49] Finally, our study was a single-center study. The study was very heterogenous and patient numbers were relatively small, so large epidemiologic study may be required to validate the findings.

In summary, the single-center study in China demonstrated that RDW was an independent predictor for mortality in obstetric critical care patients. Combining RDW and the APACHE-II score improved prognostic accuracy compared with the APACHE-II score model alone.

Contributorship statement

Yufeng Chu and Hongsheng Ren conceived of the study, and participated in its design and coordination. Mei Meng, Haiyan Zhou and Haiyan Zhou extracted data and participated in study design. Yufeng Chu and Zhongshang

For peer review only

Yuan performed the statistical analysis and drafted the manuscript. All authors read and approved the final manuscript.

Competing interests

The authors declare that there is no conflict of interests regarding the publication of this paper.

Funding

This study was funded by the National Natural Science Foundation of China (NSFC 81200238), the Research Foundation for Excellent Young and Middle-aged Scientists of Shandong Province (BS2011YY043, BS2011YY052), and the Natural Science Foundation of Shandong Province of China (ZR2013HM062).

Data sharing statement

No additional unpublished data are available.

References

1. World Health Organization. World Health Statistics 2014. Geneva, World Health Organization; 2014.
2. Zeeman GG. Obstetric critical care: A blueprint for improved outcomes. Crit Care Med 2006; 34:S208–S214.
3. Soubra SH, Guntupalli KK. Critical illness in pregnancy: an overview. Crit Care Med 2005;33(10 suppl):S248-S255.

For peer review only

4. Scarpinato L, Gerber D. Critically ill obstetrical patients: outcome and predictability. Crit Care Med 1995;23(8):1449-1451.

5. Gopalan PD, Muckart DJ. The critically ill obstetric patient: what's the score? Int J Obstet Anesth 2004;13(3):144-145.

6. Ramsay MM, James D, Steer P, et al, editors. Normal values in pregnancy. London: W.B. Saunders; 2000.

7. Lapinsky SE, Hallett D, Collop N, et al. Evaluation of standard and modified severity of illness scores in the obstetric patient. J Crit Care 2011 Oct;26(5):535.e1-7.

8. Harrison D, Penny J, Yentis S, et al. Case mix, outcome and activity for obstetric admissions to adult, general critical care units: A secondary analysis of the ICNARC Case Mix Programme Database. Crit Care 2005; 9(Suppl 3):S25–S37.

9. Paternina-Caicedo A, Rojas-Suarez J, Duenas-Castel C, et al. Mortality risk prediction with an updated Acute Physiology and Chronic Health Evaluation II Score in critically ill obstetric patients: A cohort study. J Intensive Care Med 2015 Feb;30(2):97-102.

10. Perkins SL. Examination of blood and bone marrow. In: Greer JP, Foerster J, Lukens JN, Rodgers GM, Paraksevas F, Glader BE, eds. Wintrobe's Clinical Hematology. 11th ed. Salt Lake City, Utah: Lippincott Wilkins & Williams; 2003:5–25.

For peer review only

11. McKenzie SD. Introduction to anemia. In: McKenzie SD, ed. Clinical Laboratory Hematology. Saddle River, NJ: Pearson Prentice-Hall; 2003:161–188.

12. Tonelli M, Sacks F, Arnold M, et al. For the Cholesterol and Recurrent Events (CARE) Trial InvestigatorsCirculation. Relation Between Red Blood Cell Distribution Width and Cardiovascular Event Rate in People With Coronary Disease. Circulation 2008;117:163–8.

13. Felker GM, Allen LA, Pocock S, et al. CHARM Investigators: Red cell distribution width as a novel prognostic marker in heart failure: data from the CHARM Program and the Duke Databank for Cardiovascular Diseases. J Am Coll Cardiol 2007;50:40-47.

14. Förhécz Z, Gombos T, Borgulya G, et al. Red cell distribution width in heart failure: prediction of clinical events and relationship with markers of ineffective erythropoiesis, inflammation, renal function, and nutritional state. Am Heart J 2009; 158:659–66.

15. Pascual-Figal DA, Bonaque JC, Redondo B, et al. Red blood cell distribution width predicts long-term outcome regardless of anaemia status in acute heart failure patients. Eur J Heart Fail 2009;11:840–6.

16. Wang F, Pan W, Pan S, et al. Red cell distribution width as a novel predictor of mortality in ICU patients. Ann Med 2011 Feb;43(1):40-6.

17. Ani C, Ovbiagele B. Elevated red blood cell distribution width predicts mortality in persons with known stroke. J Neurol Sci 2009;277:103–8.

For peer review only

18. Purtle SW, Moromizato T, McKane CK, et al. The association of red cell distribution width at hospital discharge and out-of-hospital mortality following critical illness. Crit Care Med 2014 Apr;42(4):918-29.

19. Kim CH, Park JT, Kim EJ, et al. An increase in red blood cell distribution width from baseline predicts mortality in patients with severe sepsis or septic shock. Crit Care 2013 Dec 9;17(6):R282.

20. Mehta RL, Kellum JA, Shah SV, et al. Acute kidney injury network: report of an initiative to improve outcomes in acute kidney injury. Crit Care 2007;11: R31.

21. Youden WJ: Index for rating diagnostic tests. Cancer 1950;3:32–5.

22. DeLong ER, DeLong DM, Clarke-Pearson DL. Comparing the areas under two or more correlated receiver operating characteristic curves: a nonparametric approach. Biometrics 1988 Sep;44(3):837-45.

23. Weiss G, Goodnough LT. Anemia of chronic disease. N Engl J Med 2005;352:1011–1023.

24. Sabina Hunziker, Leo A Celi, Joon Lee, et al. Red cell distribution width improves the simplified acute physiology score for risk prediction in unselected critically ill patients. Critical Care 2012,16:R89.

25. Heidi S. Bazick, Domingo Chang, Karthik Mahadevappa, et al. Red cell distribution width and all-cause mortality in critically ill patients. Crit Care Med 2011;39:1913–1921.

For peer review only

26. Knaus WA, Draper EA, Wagner DP, et al. APACHE II: a severity of disease classification system. Crit Care Med 1985;13:818–29.

27. Hazelgrove JF, Price C, Pappachan VJ, et al. Multicenter study of obstetric admissions to 14 intensive care units in southern England. Crit Care Med 2001 Apr;29(4):770-5.

28. Harrison D, Penny JA, Yentis SM, et al. Case mix, outcome and activity for obstetric admissions to adult, general critical care units: a secondary analysis of the ICNARC case mix programme database. Critical Care 2005;9(suppl 3):S25-S37.

29. Arabi Y, Goraj R, Horsfall D. Scoring systems in obstetric patients requiring intensive care in Saudi Arabia: a ten year review. Chest 1999;116:286S.

30. Duffy TP. Approach to the patient with anemia. In: Kelley WN, Dupont HL, Glick JH, Harris ED, Hathaway DR, Hazzard WR, Holmes EW, Hudson LD, Humes HD, Paty DW, eds. Kelley's Textbook of Internal Medicine. 3rd ed. Philadelphia, Pa: Lippincott Williams and Wilkins; 1997:1295–1299.

31. Forhecz Z, Gombos T, Borgulya G, et al. Red cell distribution width in heart failure: prediction of clinical events and relationship with markers of ineffective erythropoiesis, inflammation, renal function, and nutritional state. Am Heart J 2009, 158:659-666.

For peer review only

32. Ridker PM, Rifai N, Clearfield M, et al. Measurement of C-reactive protein for the targeting of statin therapy in the primary prevention of acute coronary events. N Engl J Med 2001;344:1959 –1965.

33. Lippi G, Targher G, Montagnana M, et al. Relation between red blood cell distribution width and inflammatory biomarkers in a large cohort of unselected outpatients. Arch Pathol Lab Med 2009;133:628–632.

34. Sesso HD, Buring JE, Rifai N, et al. C-reactive protein and the risk of developing hypertension. JAMA 2003;290:2945–2951.

35. Kolls JK. Oxidative stress in sepsis: A redox redux. J Clin Invest 2006;116:860–863.

36. Ghaffari S. Oxidative stress in the regulation of normal and neoplastic hematopoiesis. Antioxid Redox Signal 2008;10:1923–1940.

37. Salvagno GL, Sanchis-Gomar F, Picanza A. Lippi G. Red blood cell distribution width: A simple parameter with multiple clinical applications. Crit Rev Clin Lab Sci.2014 Dec 23:1-20.

38. Rojas-Suarez J, Paternina-Caicedo AJ, Miranda J, et al. Comparison of severity-of-illness scores in critically ill obstetric patients: a 6-year retrospective cohort. Crit Care Med 2014;42:1047–1054.

39. Hazelgrove JF, Price C, Pappachan VJ, et al. Multicenter study of obstetric admissions to 14 intensive care units in southern England. Crit Care Med 2001 Apr;29(4):770-5.

For peer review only

40. Karnad DR, Lapsia V, Krishnan A, et al. Prognostic factors in obstetric patients admitted to an Indian intensive care unit. Crit Care Med 2004 Jun;32(6):1294-9.

41. Higgins TL, Teres D, Copes W. Updated Mortality Probability Model (MPM-III). Chest 2005;128(4 Suppl):348S.

42. Knaus WA, Draper EA, Wagner DP, et al. APACHE II: A severity of disease classification system. Crit Care Med 1985;13:818–829.

43. Le Gall JR, Lemeshow S, Saulnier F. A new Simplified Acute Physiology Score (SAPS II) based on a European/North American multicenter study. JAMA 1993;270:2957–2963.

44. Lemeshow S, Teres D, Klar J, et al. Mortality Probability Models (MPM II) based on an international cohort of intensive care unit patients. JAMA 1993;270:2478–2486.

45. Metnitz PG, Moreno RP, Almeida E, et al. SAPS 3 Investigators: SAPS 3–From evaluation of the patient to evaluation of the intensive care unit. Part 1: Objectives, methods and cohort description. Intensive Care Med 2005;31:1336–1344.

46. Moreno RP, Metnitz PG, Almeida E, et al. SAPS 3 Investigators: SAPS 3–From evaluation of the patient to evaluation of the intensive care unit. Part 2: Development of a prognostic model for hospital mortality at ICU admission. Intensive Care Med 2005;31: 1345–1355.

For peer review only

47. Higgins TL, Kramer AA, Nathanson BH, et al. Prospective validation of the intensive care unit admission Mortality Probability Model (MPM0-III). Crit Care Med 2009;37:1619–1623.

48. Stevens TA, Carroll MA, Promecene PA, et al. Utility of acute physiology, age, and chronic health evaluation (APACHE III) score in maternal admissions to the intensive care unit. Am J Obstet Gynecol 2006;194(5):e13-e15.

49. el-Solh AA, Grant BJ. A comparison of severity of illness scoring systems for critically ill obstetric patients. Chest 1996;110(5): 1299-1304.

For peer review only

Table 1. Baseline clinical and laboratory characteristics by tertile of RDW at critical care initiation

	Tertile I	Tertile II	Tertile III	*P*-value
**RDW (%)**	10.7-13.9	13.9-15.6	15.6-20.4	<0.001
N	131	126	119	-
**Age (years)**	29.6 ± 4.3	29.3 ± 5.6	29.6 ± 5.9	0.84
**Gestational age (weeks)**	35 (32-37)	36 (33-39)	36 (32-38)	0.327
**Primary diagnosis to ICU admission (*n*, %)**				
Hypertensive disorder of pregnancy	50(38.17%)	55(43.65%)	52(43.70%)	0.417
HELLP Syndrome	12(9.16%)	10(7.94%)	8(6.72%)	0.322
Acute fatty liver of pregnancy	10(7.63%)	8(6.35%)	7(5.88%)	0.485
Obstetric sepsis	5(3.82%)	4(3.17%)	3(2.52%)	0.657
Cardiovascular disease	42 (32.06%)	31 (24.60%)	26 (21.85%)	0.162
Gastrointestinal disease	2 (1.53%)	5 (3.97%)	4 (3.36%)	0.476
Stroke	2 (1.53%)	3 (2.38%)	0 (0)	0.332
Pulmonary embolism	1 (0.76%)	0 (0)	1 (0.84%)	0.766
Others	7(5.34%)	10(7.94%)	18(15.13)	0.142
**Hemoglobin (g/L)**	109.5 ± 19.7	100.1 ± 20.7	92.5 ± 24.1	<0.001
**MCV(fL)**	89.8 ± 5.1	86.7 ± 6.0	83.6 ± 11.4	<0.001
**HCT (%)**	32.6 ± 5.8	30.5 ± 6.3	28.8 ± 6.9	<0.001
**APACHE II score (points)**	2 (2-6)	5 (3-6)	10 (6-22)	0.017
**AKI (*n*, %)**	14 (10.69%)	17 (13.49%)	14 (11.76%)	0.207
**TLSH (days)**	8 (7-12)	8 (6-13)	8 (7-12)	0.203
**ICU Mortality (*n*, %)**	0 (0%)	5 (3.97%)	15 (12.61%)	<0.001

For peer review only

RDW, red cell distribution width; MCV, mean corpuscular volume; HCT, hematocrit; APACHE-II score, Acute Physiology and Chronic Health Evaluation II score; AKI, acute kidney injury; TLSH, total length of stay in hospital. Values are presented as mean ± standard deviations or number (percentage).

For peer review only

Table 2. Univariate odds ratios of variables for predicting mortality

Variables	Odds ratio	95% CI	*P*-value
Age (years)	1.048	0.969-1.133	0.244
Hemoglobin (g/L)	0.997	0.977-1.017	0.763
MCV (fL)	0.962	0.919-1.006	0.098
Hematocrit (%)	0.997	0.934-1.064	0.929
APACHE-II score (points)	1.192	1.124-1.265	< 0.001
AKI (%)	16.61	6.580-42.014	< 0.001
TLSH (days)	0.803	0.691-0.933	0.004
gestational age (weeks)	1.023	0.920-1.138	0.677
RDW (%)	1.309	1.150-1.489	<0.001

CI, confidence interval; RDW, red cell distribution width; MCV, mean corpuscular volume; HCT, hematocrit; APACHE-II score, Acute Physiology and Chronic Health Evaluation II score; AKI, acute kidney injury; TLSH, total length of stay in hospital.

For peer review only

Table 3. Independent predictors of mortality by multivariate logistic regression analysis

Variables	Odds ratio	95% CI	*P* value
APACHE-II (points)	1.189	1.071-1.319	0.001
AKI (%)	23.784	6.129-92.296	<0.001
RDW (%)	1.401	1.156-1.697	0.001

Independent variables include age, hemoglobin, MCV, hematocrit, APACHE-II score, AKI, TLSH, gestational age, and RDW. Due to the high correlation between hemoglobin and hematocrit, hemoglobin was first regressed on hematocrit; and placed the residual and hematocrit in the multivariate regression. RDW, red cell distribution width; MCV, mean corpuscular volume; HCT, hematocrit; APACHE-II score, Acute Physiology and Chronic Health Evaluation II score; AKI, acute kidney injury; TLSH, total length of stay in hospital.

For peer review only

Figure 1 ROC curve for Acute Physiology and Chronic Health Evaluation (APACHE) II score, red blood cell distribution width (RDW) and the combination of both in predicting ICU mortality.

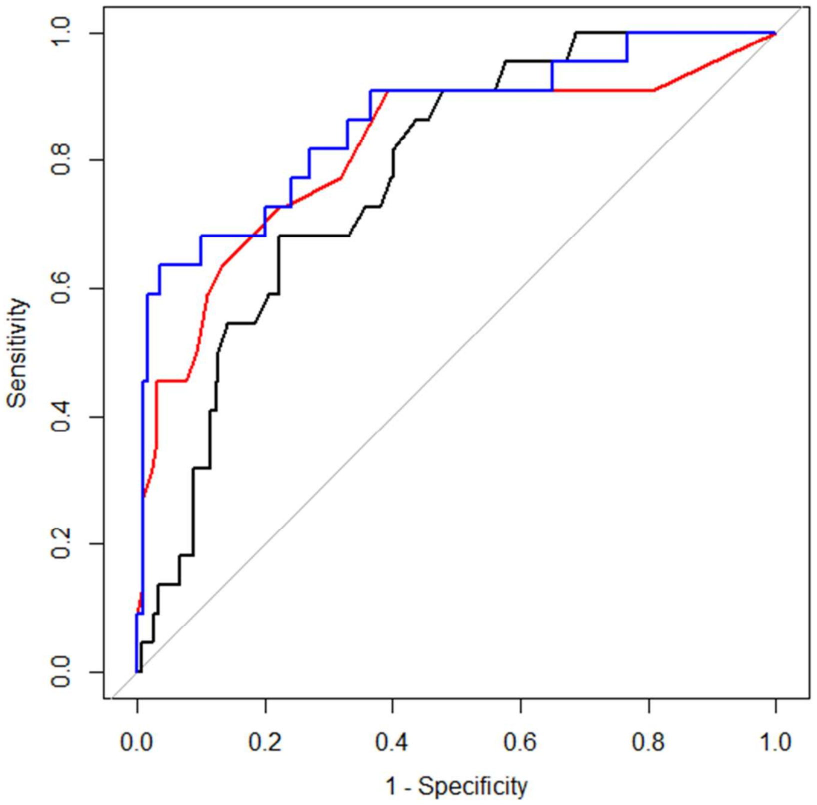

— RDW (AUC 0.752 ± 0.062)

— APACHE-II score (AUC 0.766 ± 0.031)

— RDW+ APACHE-II score (AUC 0.872 ± 0.055)

RDW, red cell distribution width; MCV, mean corpuscular volume; HCT, hematocrit; APACHE-II score, Acute Physiology and Chronic Health Evaluation II score; AKI, acute kidney injury; TLSH, total length of stay in hospital.

For peer review only - http://bmjopen.bmj.com/site/about/guidelines.xhtml

BMJ Open

Clinical Usefulness of Red Blood Cell Distribution Width in Obstetric Patients admitted to Intensive Care Unit

Journal:	*BMJ Open*
Manuscript ID	bmjopen-2016-012849.R1
Article Type:	Research
Date Submitted by the Author:	20-Dec-2016

Complete List of Authors:	Chu, Yufeng; Shandong Provincial Hospital Affiliated to Shandong University<br>Yuan, Zhongshang; Shandong University, Department of Epidemiology and Biostatistics<br>Meng, Mei; Shandong Provincial Hospital Affiliated to Shandong University<br>Zhou, Haiyan; Shandong Tumor Hospital and Institute<br>Wang, Chunting; Shandong Provincial Hospital Affiliated to Shandong University<br>Ren, Hongsheng; Shandong Provincial Hospital Affiliated to Shandong University, Department of Intensive Care Unit

**Primary Subject Heading**:	Intensive care
Secondary Subject Heading:	Obstetrics and gynaecology
Keywords:	APACHE-II score, Critical care, Mortality, OBSTETRICS, Red cell distribution width

SCHOLARONE™ Manuscripts

For peer review only

TITLE PAGE

Clinical Usefulness of Red Blood Cell Distribution Width in Obstetric Patients admitted to Intensive Care Unit

Yufeng Chu, [a, *] Zhongshang Yuan, [b, *] Mei Meng, [a] Haiyan Zhou, [c] Chunting Wang,[a] Hongsheng Ren [a]

[a] *Department of Intensive Care Unit, Shandong Provincial Hospital Affiliated to Shandong University, Shandong University, Jinan, P.R. China*

[b] *Department of Epidemiology and Biostatistics, School of Public Health, Shandong University, Jinan, P.R. China*

[c] *Department of Medical Oncology ,Shandong Tumor Hospital, Jinan, P.R. China*

Yufeng Chu and Zhongshang Yuan are co-first authors.

Corresponding author:

Hongsheng Ren, MD, Ph. D.

Department of Intensive Care Unit

Shandong Provincial Hospital Affiliated to Shandong University

No. 324 Jingwu Road, Jinan, Shandong, 250012, P.R. China

Email: sdslicu@163.com

For peer review only

Abstract

Background: Red blood cell distribution width (RDW) has been shown to predict mortality in critically ill patients. The primary aim of this study was to examine the association between RDW and hospital mortality in obstetric critical care patients. The secondary aim was to investigate whether adding RDW to Acute Physiology and Chronic Health Evaluation II (APACHE II) improved the prognostic performance of APACHE II.

Methods: This is a single center, retrospective observational study. A total of 447 consecutive obstetric patients were included. Patients were excluded if they had known hematologic diseases, history of recent blood transfusion, and patients who died or were discharged from ICU within 24 hours of admission. Each patient's ICU entrance characteristics were retrieved. We modeled the association between RDW and mortality using multivariate logistic regression. The receiver operating characteristic curve was used to examine the performance of APACHE II score and RDW in predicting mortality.

Results: A total of 376 patients were included in this present study. The hospital mortality and maternal mortality were both 5.32%. A significant association was found between baseline RDW levels and hospital or maternal mortality (hazard ratio per percent increase in RDW, 1.21; 95% CI, 1.02 to 1.57). After categorization based on tertile of baseline RDW, as well as further adjustment for hematocrit and other risk factors, a graded independent association between RDW and mortality was observed (P<0.001). The

For peer review only - http://bmjopen.bmj.com/site/about/guidelines.xhtml

addition of RDW to the APACHE II score improved the AUC for hospital or maternal mortality from 0.766 to 0.872 (P<0.001).

Conclusions: RDW is an independent predictor for hospital and maternal mortality in obstetric critical care patients. Combining RDW to APACHE II scores could further improve its prognostic performance.

Key words: APACHE-II score; Critical care; Mortality; Obstetrics; Red cell distribution width

Strengths and limitations of this study

- To our knowledge, this is the first report of red cell distribution width (RDW) as an independent prognostic outcome predictor in obstetric critical care patients.
- Since RDW is easy to obtain and highly reproducible, RDW has the potentially clinical utility to predict outcome for obstetric critical care patients.
- The study was a single-center study. It was very heterogenous, and patient numbers were relatively small, so large epidemiologic study might be required to validate the findings.

For peer review only - http://bmjopen.bmj.com/site/about/guidelines.xhtml

Introduction

Despite the advances in the care of obstetric patients, maternal mortality ratio (MMR) has remained high, especially in developing countries.[1, 2] The use of scoring systems to assess severity and predict mortality may help identify obstetric patients who truly require intensive care.[3] Acute Physiology and Chronic Health Evaluation II (APACHE II) score is a predictive scores for mortality that are widely used in ICUs. However, results from obstetric patients have shown some inconsistencies; wherein, some studies have shown that these scores are a good predictor of illness severity, while more studies have shown that these scores overestimated mortality.[4, 5, 6, 7-9] Further work is needed to improve the prognostic accuracy of APACHE II scores in obstetric patients.

Red cell distribution width (RDW) is a parameter that is easy and inexpensive to obtain, and reflects the degree of heterogeneity of erythrocyte volume.[10] RDW was used in the differential diagnosis of anemia in the past decades.[11] Recently, RDW has been shown to be a novel, independent prognostic marker for mortality, mainly in patients with cardiovascular disease and strokes, as well as in critically ill patients.[12-19] However, few ICU subpopulations have been studied so far. The obstetric population is of special interest because of the limitations of existing scoring system, which largely are based on physiological parameters.

Therefore, we conducted a retrospective study to evaluate the prognostic value of RDW, and investigated whether adding RDW could improve the prognostic performance of APACHE-II scores in critically ill obstetric patients.

For peer review only - http://bmjopen.bmj.com/site/about/guidelines.xhtml

Methods

This study was approved by the Institutional Review Board of the Provincial Hospital Affiliated to Shandong University. Obstetric patients consecutively admitted in the ICU for at least 24 hours from January 1, 2008 to December 31, 2013 were included in this retrospective observational study. The requirement for patient consent was waived because this study did not affect the patient's clinical care, and all protected health information was deleted. Obstetric patients were defined as pregnant women or up to six weeks postpartum. This study was carried out at the Shandong Provincial Hospital Affiliated to Shandong University, Jinan, China. The hospital is a 1500-bed tertiary academic hospital with 20 ICU beds. The hospital provides primary as well as tertiary care to an ethnically and socioeconomically diverse population within Shandong province and the surrounding region. The decision to transfer patients into the ICU was made by at least one senior critical care doctor and one senior obstetric doctor. Likewise, these doctors also made decisions to discharge patients or to transfer patients to general wards. Patients were excluded if they had known hematologic diseases (including leukemia, thrombotic thrombocytopenic purpura, and other hematologic diseases) or history of recent blood transfusion (less than two weeks). According to the APACHE II scores criterion of Knaus WA, 1985, [20] the recorded value in our study was based on the most deranged reading during each patient's initial 24h in an ICU, so we excluded the patients who died or were discharged less than 24 hours.

For peer review only - http://bmjopen.bmj.com/site/about/guidelines.xhtml

Data collection

Demographic and clinical characteristics including age, gestational age, parity, pregnancy status at admission, diagnosis at entry and during ICU stay, and length of hospital stay were collected. The cesarean section was considered an emergency intervention. Diagnosis of acute kidney injury (AKI) was based on the Acute Kidney Injury Network (AKIN) criteria.[21] AKI was defined as an absolute increase in serum creatinine of more than or equal to 0.3 mg/dl, a percentage increase in serum creatinine of more than or equal to 50% (1.5 fold from baseline) within 48 hours, or reduction in urine output (documented oliguria of less than 0.5 ml/Kg per hour for more than six hours).[21] The pregnancy associated with cardiac disease include congenital and acquired heart disease during pregnancy. APACHE II scores were calculated using the worst value of 12 acute physiological variables within 24 hours of presentation. These variables included temperature, blood pressure, heart rate, respiratory rate, arterial oxygenation, arterial pH, serum sodium, serum potassium, serum creatinine, hematocrit, white blood cell count, and Glasgow Coma scores. If the patient was sedated at the time of ICU admission, the last Glasgow Coma Scale obtained prior to sedation was collected. RDW, hemoglobin level, hematocrit and mean corpuscular volume (MCV) of all patients included in this study were determined at admission using a Beckman Coulter LH-750 Hematology Analyzer (Beckman Coulter, Inc., Fullerton, California), as a part of the complete blood cell count. Normal reference range for RDW in the hospital laboratory is 10.9% to 15.4%.

Study outcomes

For peer review only - http://bmjopen.bmj.com/site/about/guidelines.xhtml

All patients were followed up during hospitalization. According to the rule of our department, all ICU patients would be followed up at 1, 6, and 12 months after discharge. The primary end points of the study were hospital mortality and maternal mortality. The primary predictor of interest was RDW measured at ICU admission.

Statistics

All continuous variables were presented as means ± standard deviations, or medians with interquartile ranges as appropriate. Categorical data were summarized as number or percentage. We divided RDW into tertiles, and compared the demographics, diagnosis, clinical characteristics, laboratory test results, and APACHE-II scores of patients using analysis of variance or Kruskal-Wallis tests for continuous variables, and Chi-square or Fisher's exact tests for categorical variables. Univariate logistic regression analysis was conducted to examine the association between mortality and each of the predictors, separately. Multivariate logistic regression was further utilized to determine independent predictors of ICU mortality after adjusting for potential confounding factors. An appealing receiver operating characteristic (ROC) curve was used to examine the performance of APACHE-II scores and RDW in predicting ICU mortality. The curve represented a sensitivity plot *versus* 1-specificity. The area under the curve (AUC) was derived from the ROC curve, and the Youden index was adopted to define the optimal cut-off value.[22] We also constructed an ROC curve for the combined APACHE-II score and RDW results for predicting ICU mortality according to the weighted sum formula derived from multivariate logistic regression: logit (mortality) = 0.162 x

For peer review only - http://bmjopen.bmj.com/site/about/guidelines.xhtml

APACHE-II score + 0.203 x RDW - 7.503; wherein, logit (mortality) is the logarithm of the odds of a critically ill obstetric patient dying in the ICU. Differences between the AUC were detected by Delong's test which was a nonparametric approach and could generate an estimated covariance matrix by using the theory on generalized U-statistics.[23] Two-sided *P*-values <0.05 were considered statistically significant, and all analyses were performed with R software (http://www.cran.r-project.org/).

Results

Population

A total of 447 obstetric patients who were admitted to ICU were initially enrolled in this study. ICU admission rate was 21.73 per 1,000 births. Eight patients who were diagnosed with hematologic disease and 59 patients who received red blood cell transfusion within two weeks were excluded. Two patients who died within 24 hours after ICU admission were also excluded. Two patients were excluded for missing data. In the final analysis, 376 patients were included in this study.

Association between RDW and hospital mortality

A total of 20 deaths occurred in this cohort during the study period. As mentioned before, all ICU patients would be followed up at 1, 6, and 12 months after discharge. As we know, of the total of 376 obstetric patients who were admitted to ICU, all the death occurred in the ICU. That is to say, there was no death outside of hospital or ICU. Thus, the maternal mortality was

For peer review only - http://bmjopen.bmj.com/site/about/guidelines.xhtml

equal to hospital mortality in this population. In this study, heart failure was the major cause of death in the cohort (n=8; 40.0%), followed by acute fatty liver of pregnancy (n=5; 25%), postpartum hemorrhage (n=2), hemorrhagic shock caused by liver tumor rupture (n=1), HELLP syndrome (n=1), acute pulmonary embolism (n=1), stroke (n=1), and liver failure caused by severe hepatitis B (n=1). The hospital mortality and maternal mortality were both 5.32%, and RDW ranged from 10.7% to 24.0% (median, 14.8%; interquartile range, 13.4% to 16.0%).

Participants were divided into three categories on the basis of their baseline tertiles of RDW level: tertile I, 10.7%-13.9%; tertile II, 13.9%-15.6%; tertile III, 15.6%-20.4% (all $P<0.001$). There was no difference in age, gestational weeks, primary reasons for ICU admission, AKI morbidity, as well as in the total length of stay in hospital (TLSH) among the three tertiles. Hb, MCV and HCT significantly decreased with the increase of RDW, while APACHE II scores and hospital mortality significantly increased with the increase of RDW (Table 1). A significant association was found between baseline RDW levels and hospital mortality (hazard ratio per percent increase in RDW, 1.21; 95% CI, 1.02 to 1.57).

Regression analysis for hospital mortality

Univariate logistic regression analysis demonstrated that patients with higher RDW, higher APACHE-II scores, higher AKI morbidity, and longer TLSH days had significantly greater death hazards (Table 2). Multivariate logistic regression analysis revealed that RDW, AKI and APACHE-II scores were independent predictors of hospital mortality (Table 3). The association of

For peer review only

For peer review only - http://bmjopen.bmj.com/site/about/guidelines.xhtml

RDW and hospital mortality remained significant after adjusting for age, hemoglobin, MCV, hematocrit, APACHE-II score and AKI. RDW was a significant outcome predictor, which is independent of APACHE-II scores.

A ROC curve was drawn to evaluate the value for RDW and APACHE-II scores in predicting mortality (Figure 1). The AUC was calculated as 0.766 (95% CI, 0.705 to 0.826) for the APACHE-II score and 0.752 (95% CI, 0.684 to 0.862) for RDW. Optimal cut-off value of the APACHE-II score for predicting mortality was five points, which gave a sensitivity of 90.9% and a specificity of 60.7%. The optimal cut-off value of RDW was 16.1%, which gave a sensitivity of 68.2% and specificity of 77.9%. To further clarify whether RDW had an additive power with the APACHE-II score for hospital mortality, we combined RDW and the APACHE-II score to draw a third ROC curve, as shown in Figure 1. Compared with the APACHE-II score, adding RDW to APACHE-II scores improved the AUC from 0.766 to 0.872 (P<0.001). These results suggested that combining RDW and APACHE-II added to the ability to discriminate mortality risk. As in multivariate logistic regression analysis, AKI was another strong predictor for hospital mortality. We conducted the ROC curve analysis using all three variables including RDW, APACHE-II and AKI. Adding AKI improved the AUC from 0.872 to 0.884 (P>0.05), however, when we compared them by Delong's test, no significance can be found.

Discussion

The main finding of our study was that RDW was independently associated with hospital and maternal mortality in obstetric critical care patients . The

For peer review only

For peer review only - http://bmjopen.bmj.com/site/about/guidelines.xhtml

association remained significant even after adjusting for APACHE-II scores, hemoglobin levels, hematocrit and mean corpuscular volume. Combining RDW and the APACHE-II score improved the prognostic accuracy of hospital mortality predicted by APACHE-II alone in the study population.

We found that RDW was independently associated with hospital and maternal mortality in a Chinese obstetric ICU population. Patients were excluded if they had a history of recent blood transfusions because RDW could be increased in anemia or after blood transfusions.[11, 24] In this present study, higher levels of RDW were associated with hospital and maternal mortality even after adjusting for hemoglobin and hematocrit. In addition, the independent association between RDW and hospital mortality or maternal mortality was not eliminated even after adjusting for other potential confounders, including mean corpuscular volume, hemoglobin, hematocrit, APACHE-II score, gestational age and AKI. Our results were consistent with previous analyses of two large populations of critically ill patients.[25, 26] Further, our results were also similar to the findings obtained in a single-center observational study in 2011; [16] wherein, the independent association between RDW and all-cause mortality in critically ill patients was shown.

The important finding in our analysis was that combining RDW and APACHE-II score performed better than APACHE-II score alone in predicting hospital mortality or maternal mortality in critically ill obstetric patients. The APACHE-II scoring system developed in 1985 has shown a positive correlation with hospital mortality, and was one of the most common models used for evaluating the severity of a disease in critically ill patients.[20] Previous

For peer review only - http://bmjopen.bmj.com/site/about/guidelines.xhtml

studies[27-29] suggested APACHE II score was a good discriminator of illness severity in obstetric cohorts. The sensitivity and specificity of the APACHE II score was evaluated with the use of receiver operating characteristic curve analysis. In accordance with previous studies[27-29], the APACHE-II score was also demonstrated to have a strong power to predict hospital mortality or maternal mortality (AUC=0.766) in the present study. Despite its moderate discriminative power alone (AUC=0.752), adding RDW to the APACHE-II score resulted to a better increase in the AUC from 0.766 to 0.872 (P<0.001). Combining RDW with APACHE-II score significantly improved prognostic performance in critically ill obstetric patients. Similarly, another study by Wang *et al.* [16] also revealed that adding RDW to APACHE-II score significantly improved prognostic reliability of APACHE-II score in identifying critically ill patients. Since RDW is a simple, inexpensive, and widely available test as part of the complete blood count, these data may have significant clinical implications for determining prognosis in critically ill obstetric patients. However, no significant improvement on prognostic performance was observed when AKI was associated with RDW and APACHE-II score, even though AKI was another strong predictor for hospital mortality in multivariate logistic regression analysis. The reason was not clear, and further study would be needed.

The pathophysiologic mechanism underlying the association of higher RDW with worse outcomes in critically ill obstetric patients remains unclear. Generally, the increase in RDW reflects either impaired erythropoiesis, abnormal red blood cell survival, or both. Metabolic abnormalities such as shortening of telomere length, poor nutritional status, [30, 31] inflammation, [32-34]

For peer review only - http://bmjopen.bmj.com/site/about/guidelines.xhtml

oxidative stress, [35, 36] dyslipidemia, hypertension, erythrocyte fragmentation, and alteration of erythropoietin function might contribute to RDW increase.[37] In normal parturition, the increase of RDW might be related to stimulus-induced reticulocytosis in the last few weeks.[29] This might not be the case in our study; wherein, even if five patients with spontaneous onset of labor were excluded, RDW remained as a significant predictor of mortality.

There were 20570 births from 2008 to 2013 in Shandong Provincial Hospital and 447 ICU admissions. A total of 28 deaths occurred in the hospital during the 6-year study period resulting in a maternal mortality rate of 136 per 100,000 births. There were 22 deaths occurred in the ICU, and the others occurred in emergency room (n=2) and maternity ward (n=4). In this present study, the admission rate of 21.73 per 1,000 births was relatively higher than those reported by other studies, [38-39] and the mean APACHE-II score was relatively lower compared with other groups.[7, 16, 40] The lack of a high dependency unit as a referral center in local areas for complicated pregnancies could partially explain these difference.

This study had several limitations. First, we did not evaluate the evolution of RDW, and thus, it remained unclear whether changes in RDW over time may provide additional prognostic information. Second, we only used the APACHE-II score to predict mortality in obstetric patients, which was limited by spontaneous improvement after delivery in peripartum women, [41-47] and inconsistent results.[48, 49] Finally, our study was a single-center study. The study was very heterogenous and patient numbers were relatively small, so large epidemiologic study may be required to validate the findings.

For peer review only - http://bmjopen.bmj.com/site/about/guidelines.xhtml

In summary, the single-center study in China demonstrated that RDW was an independent predictor for mortality in obstetric critical care patients. Combining RDW and the APACHE-II score improved prognostic accuracy compared with the APACHE-II score model alone.

Acknowledgements

This study was funded by the National Natural Science Foundation of China (NSFC 81200238), the Research Foundation for Excellent Young and Middle-aged Scientists of Shandong Province (BS2011YY043, BS2011YY052),the Young Scholars Program of Shandong University (2016WLJH23), the Clinical medicine science and technology innovation plan of Jinan Bureau of Science & Technology (201602164), the Shandong Provincial Department of Science & Techno-logy research and development plan (2016GSF201052), and the Natural Science Foundation of Shandong Province of China (ZR2013HM062).

Conflict of Interests

The authors declare that there is no conflict of interests regarding the publication of this paper.

References

1. World Health Organization. World Health Statistics 2014. Geneva, World Health Organization; 2014.

2. Zeeman GG. Obstetric critical care: A blueprint for improved outcomes. Crit Care Med 2006; 34:S208–S214.

For peer review only - http://bmjopen.bmj.com/site/about/guidelines.xhtml

3. Soubra SH, Guntupalli KK. Critical illness in pregnancy: an overview. Crit Care Med 2005;33(10 suppl):S248-S255.

4. Scarpinato L, Gerber D. Critically ill obstetrical patients: outcome and predictability. Crit Care Med 1995;23(8):1449-1451.

5. Gopalan PD, Muckart DJ. The critically ill obstetric patient: what's the score? Int J Obstet Anesth 2004;13(3):144-145.

6. Ramsay MM, James D, Steer P, et al, editors. Normal values in pregnancy. London: W.B. Saunders; 2000.

7. Lapinsky SE, Hallett D, Collop N, et al. Evaluation of standard and modified severity of illness scores in the obstetric patient. J Crit Care 2011 Oct;26(5):535.e1-7.

8. Harrison D, Penny J, Yentis S, et al. Case mix, outcome and activity for obstetric admissions to adult, general critical care units: A secondary analysis of the ICNARC Case Mix Programme Database. Crit Care 2005; 9(Suppl 3):S25–S37.

9. Paternina-Caicedo A, Rojas-Suarez J, Duenas-Castel C, et al. Mortality risk prediction with an updated Acute Physiology and Chronic Health Evaluation II Score in critically ill obstetric patients: A cohort study. J Intensive Care Med 2015 Feb;30(2):97-102.

10. Perkins SL. Examination of blood and bone marrow. In: Greer JP, Foerster J, Lukens JN, Rodgers GM, Paraksevas F, Glader BE, eds. Wintrobe's Clinical Hematology. 11th ed. Salt Lake City, Utah: Lippincott Wilkins & Williams; 2003:5–25.

For peer review only

For peer review only - http://bmjopen.bmj.com/site/about/guidelines.xhtml

11. McKenzie SD. Introduction to anemia. In: McKenzie SD, ed. Clinical Laboratory Hematology. Saddle River, NJ: Pearson Prentice-Hall; 2003:161–188.

12. Tonelli M, Sacks F, Arnold M, et al. For the Cholesterol and Recurrent Events (CARE) Trial InvestigatorsCirculation. Relation Between Red Blood Cell Distribution Width and Cardiovascular Event Rate in People With Coronary Disease. Circulation 2008;117:163–8.

13. Felker GM, Allen LA, Pocock S, et al. CHARM Investigators: Red cell distribution width as a novel prognostic marker in heart failure: data from the CHARM Program and the Duke Databank for Cardiovascular Diseases. J Am Coll Cardiol 2007;50:40-47.

14. Förhécz Z, Gombos T, Borgulya G, et al. Red cell distribution width in heart failure: prediction of clinical events and relationship with markers of ineffective erythropoiesis, inflammation, renal function, and nutritional state. Am Heart J 2009; 158:659–66.

15. Pascual-Figal DA, Bonaque JC, Redondo B, et al. Red blood cell distribution width predicts long-term outcome regardless of anaemia status in acute heart failure patients. Eur J Heart Fail 2009;11:840–6.

16. Wang F, Pan W, Pan S, et al. Red cell distribution width as a novel predictor of mortality in ICU patients. Ann Med 2011 Feb;43(1):40-6.

17. Ani C, Ovbiagele B. Elevated red blood cell distribution width predicts mortality in persons with known stroke. J Neurol Sci 2009;277:103–8.

For peer review only

For peer review only - http://bmjopen.bmj.com/site/about/guidelines.xhtml

18. Purtle SW, Moromizato T, McKane CK, et al. The association of red cell distribution width at hospital discharge and out-of-hospital mortality following critical illness. Crit Care Med 2014 Apr;42(4):918-29.

19. Kim CH, Park JT, Kim EJ, et al. An increase in red blood cell distribution width from baseline predicts mortality in patients with severe sepsis or septic shock. Crit Care 2013 Dec 9;17(6):R282.

20. Youden WJ: Index for rating diagnostic tests. Cancer 1950;3:32–5.

21. DeLong ER, DeLong DM, Clarke-Pearson DL. Comparing the areas under two or more correlated receiver operating characteristic curves: a nonparametric approach. Biometrics 1988 Sep;44(3):837-45.

22. Weiss G, Goodnough LT. Anemia of chronic disease. N Engl J Med 2005;352:1011–1023.

23. Sabina Hunziker, Leo A Celi, Joon Lee, et al. Red cell distribution width improves the simplified acute physiology score for risk prediction in unselected critically ill patients. Critical Care 2012,16:R89.

24. Heidi S. Bazick, Domingo Chang, Karthik Mahadevappa, et al. Red cell distribution width and all-cause mortality in critically ill patients. Crit Care Med 2011;39:1913–1921.

25. Knaus WA, Draper EA, Wagner DP, et al. APACHE II: a severity of disease classification system. Crit Care Med 1985;13:818–29.

26. Knaus WA, Draper EA, Wagner DP, et al. APACHE II: a severity of disease classification system. Crit Care Med 1985;13:818–29.

For peer review only

For peer review only - http://bmjopen.bmj.com/site/about/guidelines.xhtml

27. Hazelgrove JF, Price C, Pappachan VJ, et al. Multicenter study of obstetric admissions to 14 intensive care units in southern England. Crit Care Med 2001 Apr;29(4):770-5.

28. Harrison D, Penny JA, Yentis SM, et al. Case mix, outcome and activity for obstetric admissions to adult, general critical care units: a secondary analysis of the ICNARC case mix programme database. Critical Care 2005;9(suppl 3):S25-S37.

29. Arabi Y, Goraj R, Horsfall D. Scoring systems in obstetric patients requiring intensive care in Saudi Arabia: a ten year review. Chest 1999;116:286S.

30. Duffy TP. Approach to the patient with anemia. In: Kelley WN, Dupont HL, Glick JH, Harris ED, Hathaway DR, Hazzard WR, Holmes EW, Hudson LD, Humes HD, Paty DW, eds. Kelley's Textbook of Internal Medicine. 3rd ed. Philadelphia, Pa: Lippincott Williams and Wilkins; 1997:1295–1299.

31. Forhecz Z, Gombos T, Borgulya G, et al. Red cell distribution width in heart failure: prediction of clinical events and relationship with markers of ineffective erythropoiesis, inflammation, renal function, and nutritional state. Am Heart J 2009, 158:659-666.

32. Ridker PM, Rifai N, Clearfield M, et al. Measurement of C-reactive protein for the targeting of statin therapy in the primary prevention of acute coronary events. N Engl J Med 2001;344:1959 –1965.

For peer review only

33. Lippi G, Targher G, Montagnana M, et al. Relation between red blood cell distribution width and inflammatory biomarkers in a large cohort of unselected outpatients. Arch Pathol Lab Med 2009;133:628–632.

34. Sesso HD, Buring JE, Rifai N, et al. C-reactive protein and the risk of developing hypertension. JAMA 2003;290:2945–2951.

35. Kolls JK. Oxidative stress in sepsis: A redox redux. J Clin Invest 2006;116:860–863.

36. Ghaffari S. Oxidative stress in the regulation of normal and neoplastic hematopoiesis. Antioxid Redox Signal 2008;10:1923–1940.

37. Salvagno GL, Sanchis-Gomar F, Picanza A. Lippi G. Red blood cell distribution width: A simple parameter with multiple clinical applications. Crit Rev Clin Lab Sci.2014 Dec 23:1-20.

38. Rojas-Suarez J, Paternina-Caicedo AJ, Miranda J, et al. Comparison of severity-of-illness scores in critically ill obstetric patients: a 6-year retrospective cohort. Crit Care Med 2014;42:1047–1054.

39. Hazelgrove JF, Price C, Pappachan VJ, et al. Multicenter study of obstetric admissions to 14 intensive care units in southern England. Crit Care Med 2001 Apr;29(4):770-5.

40. Karnad DR, Lapsia V, Krishnan A, et al. Prognostic factors in obstetric patients admitted to an Indian intensive care unit. Crit Care Med 2004 Jun;32(6):1294-9.

For peer review only

For peer review only - http://bmjopen.bmj.com/site/about/guidelines.xhtml

41. Higgins TL, Teres D, Copes W. Updated Mortality Probability Model (MPM-III). Chest 2005;128(4 Suppl):348S.

42. Knaus WA, Draper EA, Wagner DP, et al. APACHE II: A severity of disease classification system. Crit Care Med 1985;13:818–829.

43. Le Gall JR, Lemeshow S, Saulnier F. A new Simplified Acute Physiology Score (SAPS II) based on a European/North American multicenter study. JAMA 1993;270:2957–2963.

44. Lemeshow S, Teres D, Klar J, et al. Mortality Probability Models (MPM II) based on an international cohort of intensive care unit patients. JAMA 1993;270:2478–2486.

45. Metnitz PG, Moreno RP, Almeida E, et al. SAPS 3 Investigators: SAPS 3–From evaluation of the patient to evaluation of the intensive care unit. Part 1: Objectives, methods and cohort description. Intensive Care Med 2005;31:1336–1344.

46. Moreno RP, Metnitz PG, Almeida E, et al. SAPS 3 Investigators: SAPS 3–From evaluation of the patient to evaluation of the intensive care unit. Part 2: Development of a prognostic model for hospital mortality at ICU admission. Intensive Care Med 2005;31: 1345–1355.

47. Higgins TL, Kramer AA, Nathanson BH, et al. Prospective validation of the intensive care unit admission Mortality Probability Model (MPM0-III). Crit Care Med 2009;37:1619–1623.

48. Stevens TA, Carroll MA, Promecene PA, et al. Utility of acute physiology, age, and chronic health evaluation (APACHE III) score in maternal

For peer review only - http://bmjopen.bmj.com/site/about/guidelines.xhtml

admissions to the intensive care unit. Am J Obstet Gynecol 2006;194(5):e13-e15.

49. el-Solh AA, Grant BJ. A comparison of severity of illness scoring systems for critically ill obstetric patients. Chest 1996;110(5): 1299-1304.

For peer review only

For peer review only - http://bmjopen.bmj.com/site/about/guidelines.xhtml

Table 1. Baseline clinical and laboratory characteristics by tertile of RDW at critical care initiation

	Tertile I	Tertile II	Tertile III	*P*-value
**RDW (%)**	10.7-13.9	13.9-15.6	15.6-20.4	<0.001
N	131	126	119	-
**Age (years)**	29.6 ± 4.3	29.3 ± 5.6	29.6 ± 5.9	0.84
**Gestational age (weeks)**	35 (32-37)	36 (33-39)	36 (32-38)	0.327
**Primary diagnosis to ICU admission (*n*, %)**				
Hypertensive disorder of pregnancy	50(38.17%)	55(43.65%)	52(43.70%)	0.417
HELLP Syndrome	12(9.16%)	10(7.94%)	8(6.72%)	0.322
Acute fatty liver of pregnancy	10(7.63%)	8(6.35%)	7(5.88%)	0.485
Obstetric sepsis	5(3.82%)	4(3.17%)	3(2.52%)	0.657
Cardiovascular disease	42 (32.06%)	31 (24.60%)	26 (21.85%)	0.162
Gastrointestinal disease	2 (1.53%)	5 (3.97%)	4 (3.36%)	0.476
Stroke	2 (1.53%)	3 (2.38%)	0 (0)	0.332
Pulmonary embolism	1 (0.76%)	0 (0)	1 (0.84%)	0.766
Others	7(5.34%)	10(7.94%)	18(15.13)	0.142
**Hemoglobin (g/L)**	109.5 ± 19.7	100.1 ± 20.7	92.5 ± 24.1	<0.001
**MCV(fL)**	89.8 ± 5.1	86.7 ± 6.0	83.6 ± 11.4	<0.001
**HCT (%)**	32.6 ± 5.8	30.5 ± 6.3	28.8 ± 6.9	<0.001
**APACHE II score (points)**	2 (2-6)	5 (3-6)	10 (6-22)	0.017
**AKI (*n*, %)**	14 (10.69%)	17 (13.49%)	14 (11.76%)	0.207
**TLSH (days)**	8 (7-12)	8 (6-13)	8 (7-12)	0.203
**Hospital Mortality (*n*, %)**	0 (0%)	5 (3.97%)	15 (12.61%)	<0.001

For peer review only

For peer review only - http://bmjopen.bmj.com/site/about/guidelines.xhtml

RDW, red cell distribution width; MCV, mean corpuscular volume; HCT, hematocrit; APACHE-II score, Acute Physiology and Chronic Health Evaluation II score; AKI, acute kidney injury; TLSH, total length of stay in hospital. Values are presented as mean ± standard deviations or number (percentage).

For peer review only

For peer review only - http://bmjopen.bmj.com/site/about/guidelines.xhtml

467 **Table 2. Univariate odds ratios of variables for predicting mortality**

Variables	Odds ratio	95% CI	*P*-value
Age (years)	1.048	0.969-1.133	0.244
Hemoglobin (g/L)	0.997	0.977-1.017	0.763
MCV (fL)	0.962	0.919-1.006	0.098
Hematocrit (%)	0.997	0.934-1.064	0.929
APACHE-II score (points)	1.192	1.124-1.265	< 0.001
AKI (%)	16.61	6.580-42.014	< 0.001
TLSH (days)	0.803	0.691-0.933	0.004
gestational age (weeks)	1.023	0.920-1.138	0.677
RDW (%)	1.309	1.150-1.489	<0.001

468

469 CI, confidence interval; RDW, red cell distribution width; MCV, mean
470 corpuscular volume; HCT, hematocrit; APACHE-II score, Acute Physiology
471 and Chronic Health Evaluation II score; AKI, acute kidney injury; TLSH, total
472 length of stay in hospital.

473

For peer review only - http://bmjopen.bmj.com/site/about/guidelines.xhtml

Table 3. Independent predictors of mortality by multivariate logistic regression analysis

Variables	Odds ratio	95% CI	*P* value
APACHE-II (points)	1.189	1.071-1.319	0.001
AKI (%)	23.784	6.129-92.296	<0.001
RDW (%)	1.401	1.156-1.697	0.001

Independent variables include age, hemoglobin, MCV, hematocrit, APACHE-II score, AKI, TLSH, gestational age, and RDW. Due to the high correlation between hemoglobin and hematocrit, hemoglobin was first regressed on hematocrit; and placed the residual and hematocrit in the multivariate regression. RDW, red cell distribution width; MCV, mean corpuscular volume; HCT, hematocrit; APACHE-II score, Acute Physiology and Chronic Health Evaluation II score; AKI, acute kidney injury; TLSH, total length of stay in hospital.

For peer review only - http://bmjopen.bmj.com/site/about/guidelines.xhtml

Figure 1 ROC curve for Acute Physiology and Chronic Health Evaluation (APACHE) II score, red blood cell distribution width (RDW) and the combination of both in predicting hospital mortality.

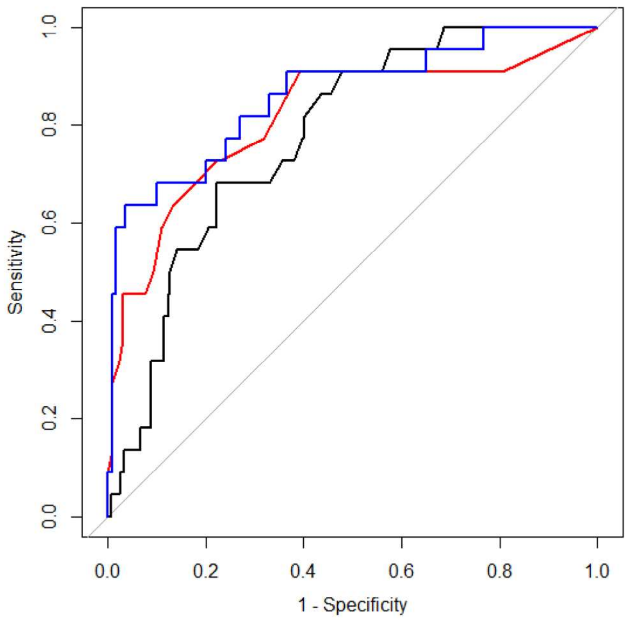

– RDW (AUC 0.752 ± 0.062)

– APACHE-II score (AUC 0.766 ± 0.031)

– RDW+ APACHE-II score (AUC 0.872 ± 0.055)

RDW, red cell distribution width; MCV, mean corpuscular volume; HCT, hematocrit; APACHE-II score, Acute Physiology and Chronic Health Evaluation II score; AKI, acute kidney injury; TLSH, total length of stay in hospital.

179x220mm (300 x 300 DPI)

STROBE 2007 (v4) Statement—Checklist of items that should be included in reports of *cohort studies*

Section/Topic	Item #	Recommendation	Reported on page #
**Title and abstract**	1	(*a*) Indicate the study's design with a commonly used term in the title or the abstract	2
		(*b*) Provide in the abstract an informative and balanced summary of what was done and what was found	2
**Introduction**			
Background/rationale	2	Explain the scientific background and rationale for the investigation being reported	4
Objectives	3	State specific objectives, including any prespecified hypotheses	4
**Methods**			
Study design	4	Present key elements of study design early in the paper	5
Setting	5	Describe the setting, locations, and relevant dates, including periods of recruitment, exposure, follow-up, and data collection	6-7
Participants	6	(*a*) Give the eligibility criteria, and the sources and methods of selection of participants. Describe methods of follow-up	7
		(*b*) For matched studies, give matching criteria and number of exposed and unexposed	
Variables	7	Clearly define all outcomes, exposures, predictors, potential confounders, and effect modifiers. Give diagnostic criteria, if applicable	6-10
Data sources/ measurement	8*	For each variable of interest, give sources of data and details of methods of assessment (measurement). Describe comparability of assessment methods if there is more than one group	*6-10*
Bias	9	Describe any efforts to address potential sources of bias	5
Study size	10	Explain how the study size was arrived at	5
Quantitative variables	11	Explain how quantitative variables were handled in the analyses. If applicable, describe which groupings were chosen and why	7-8
Statistical methods	12	(*a*) Describe all statistical methods, including those used to control for confounding	7-8
		(*b*) Describe any methods used to examine subgroups and interactions	7-8
		(*c*) Explain how missing data were addressed	7-8
		(*d*) If applicable, explain how loss to follow-up was addressed	7-8
		(*e*) Describe any sensitivity analyses	7-8
**Results**			

For peer review only

Participants	13*	(a) Report numbers of individuals at each stage of study—eg numbers potentially eligible, examined for eligibility, confirmed eligible, included in the study, completing follow-up, and analysed	8-9
		(b) Give reasons for non-participation at each stage	
		(c) Consider use of a flow diagram	
Descriptive data	14*	(a) Give characteristics of study participants (eg demographic, clinical, social) and information on exposures and potential confounders	8-10
		(b) Indicate number of participants with missing data for each variable of interest	
		(c) Summarise follow-up time (eg, average and total amount)	
Outcome data	15*	Report numbers of outcome events or summary measures over time	8-10
Main results	16	(*a*) Give unadjusted estimates and, if applicable, confounder-adjusted estimates and their precision (eg, 95% confidence interval). Make clear which confounders were adjusted for and why they were included	8-10
		(*b*) Report category boundaries when continuous variables were categorized	8-10
		(*c*) If relevant, consider translating estimates of relative risk into absolute risk for a meaningful time period	
Other analyses	17	Report other analyses done—eg analyses of subgroups and interactions, and sensitivity analyses	8-10
**Discussion**			
Key results	18	Summarise key results with reference to study objectives	10-14
**Limitations**			
Interpretation	20	Give a cautious overall interpretation of results considering objectives, limitations, multiplicity of analyses, results from similar studies, and other relevant evidence	13
Generalisability	21	Discuss the generalisability (external validity) of the study results	
**Other information**			
Funding	22	Give the source of funding and the role of the funders for the present study and, if applicable, for the original study on which the present article is based	14

*Give information separately for cases and controls in case-control studies and, if applicable, for exposed and unexposed groups in cohort and cross-sectional studies.

Note: An Explanation and Elaboration article discusses each checklist item and gives methodological background and published examples of transparent reporting. The STROBE checklist is best used in conjunction with this article (freely available on the Web sites of PLoS Medicine at http://www.plosmedicine.org/, Annals of Internal Medicine at http://www.annals.org/, and Epidemiology at http://www.epidem.com/). Information on the STROBE Initiative is available at www.strobe-statement.org.

For peer review only

RDW as a risk factor in obstetric patients admitted to the ICU – a single center, retrospective cohort study.

Journal:	*BMJ Open*
Manuscript ID	bmjopen-2016-012849.R2
Article Type:	Research
Date Submitted by the Author:	20-Feb-2017

Complete List of Authors:	Chu, Yufeng; Shandong Provincial Hospital Affiliated to Shandong University<br>Yuan, Zhongshang; Shandong University, Department of Epidemiology and Biostatistics<br>Meng, Mei; Shandong Provincial Hospital Affiliated to Shandong University<br>Zhou, Haiyan; Shandong Tumor Hospital and Institute<br>Wang, Chunting; Shandong Provincial Hospital Affiliated to Shandong University<br>Ren, Hongsheng; Shandong Provincial Hospital Affiliated to Shandong University, Department of Intensive Care Unit

**Primary Subject Heading**:	Intensive care
Secondary Subject Heading:	Obstetrics and gynaecology
Keywords:	APACHE-II score, Critical care, Mortality, OBSTETRICS, Red cell distribution width

only

TITLE PAGE

RDW as a risk factor in obstetric patients admitted to the ICU – a single center, retrospective cohort study.

Yufeng Chu,[a,*] Zhongshang Yuan,[b,*] Mei Meng,[a] Haiyan Zhou,[c] Chunting Wang,[a] Hongsheng Ren[a]

[a]*Department of Intensive Care Unit, Shandong Provincial Hospital Affiliated to Shandong University, Shandong University, Jinan, P.R. China*

[b]*Department of Epidemiology and Biostatistics, School of Public Health, Shandong University, Jinan, P.R. China*

[c]*Department of Medical Oncology ,Shandong Tumor Hospital, Jinan, P.R. China*

Yufeng Chu and Zhongshang Yuan are co-first authors.

Corresponding author:

Hongsheng Ren, MD, Ph. D.

Department of Intensive Care Unit

Shandong Provincial Hospital Affiliated to Shandong University

No. 324 Jingwu Road, Jinan, Shandong, 250012, P.R. China

Email: sdslicu@163.com

For peer review only - http://bmjopen.bmj.com/site/about/guidelines.xhtml

Abstract

Background: Red blood cell distribution width (RDW) has been shown to predict mortality in critically ill patients. The primary aim of this study was to examine the association between RDW and hospital mortality in obstetric critical care patients. The secondary aim was to investigate whether adding RDW to Acute Physiology and Chronic Health Evaluation II (APACHE II) improved the prognostic performance of APACHE II.

Methods: This is a single center, retrospective observational study. Patients were excluded if they had known hematologic diseases, history of recent blood transfusion, and patients who died or were discharged from ICU within 24 hours of admission were also excluded. Each patient's ICU entrance characteristics were retrieved. Multivariate logistic regression was used to determine the association between RDW and mortality. The receiver operating characteristic curve was used to examine the performance of APACHE II score and RDW in predicting mortality.

Results: A total of 376 patients were included in this present study and the hospital mortality was 5.32%. A significant association was found between baseline RDW levels and hospital mortality (odds ratio per percent increase in RDW, 1.21; 95% CI, 1.02 to 1.57). After categorization based on tertile of baseline RDW, as well as further adjustment for hematocrit and other risk factors, a graded independent association between RDW and mortality was observed (*P*<0.001). The addition of RDW to the APACHE II score improved the AUC for hospital mortality from 0.766 to 0.872 (*P*<0.001).

For peer review only - http://bmjopen.bmj.com/site/about/guidelines.xhtml

Conclusions: RDW is an independent predictor for hospital mortality in obstetric critical care patients. Combining RDW to APACHE II scores could further improve its prognostic performance.

Key words: APACHE-II score; Critical care; Mortality; Obstetrics; Red cell distribution width

Strengths and limitations of this study

- To our knowledge, this is the first report of red cell distribution width (RDW) as an independent prognostic outcome predictor in obstetric critical care patients.
- The advantage of RDW is that it is easy to obtain and highly reproducible, therefore, RDW may have the potential clinical utility to predict outcome for obstetric critical care patients.
- Results discussed in this study are coming from a single-center with relatively small patient numbers and high variability. A larger population based cohort study might greatly strengthen our findings.

For peer review only

Introduction

Maternal mortality ratio (MMR) remain high despite the advances in the critical care of obstetric patients, especially in developing countries.[1, 2] The use of scoring systems to assess severity and predict mortality may help identify obstetric patients who truly require intensive care.[3] Acute Physiology and Chronic Health Evaluation II (APACHE II) score is a predictive scores for mortality that are widely used in ICUs. However, results from obstetric patients were inconsistencies; wherein, some studies have shown that these scores are a good predictor of illness severity, more recent studies have shown that these scores overestimated mortality.[4, 5, 6, 7-9] Further work is needed to improve the prognostic accuracy of APACHE II scores in obstetric patients.

Red cell distribution width (RDW) is a parameter that is easy and inexpensive to obtain, and reflects the degree of heterogeneity of erythrocyte volume.[10] RDW was used in the differential diagnosis of anemia in the past decades.[11] Recently, RDW has been shown to be a novel, independent prognostic marker for mortality, mainly in patients with cardiovascular disease and strokes, as well as in critically ill patients.[12-19] However, few ICU subpopulations have been studied so far. The obstetric population is of special interest because of the limitations of existing scoring system, which are largely based on physiological parameters.

Therefore, we conducted a retrospective study to evaluate the prognostic value of RDW, and investigated whether adding RDW could improve the prognostic performance of APACHE-II scores in critically ill obstetric patients.

For peer review only - http://bmjopen.bmj.com/site/about/guidelines.xhtml

For peer review only

Methods

This study was approved by the Institutional Review Board of the Provincial Hospital Affiliated to Shandong University. Obstetric patients consecutively admitted in the ICU for at least 24 hours from January 1, 2008 to December 31, 2013 were included in this retrospective observational study. The requirement for patient consent was waived because this study did not affect the patient's clinical care, and all protected health information was deleted. Obstetric patients were defined as pregnant women or up to six weeks postpartum. This study was carried out at the Shandong Provincial Hospital Affiliated to Shandong University, Jinan, China. The hospital is a 1500-bed tertiary academic hospital with 20 ICU beds. The hospital provides primary as well as tertiary care to an ethnically and socioeconomically diverse population within Shandong province and the surrounding region. The decision to transfer patients into the ICU was made by at least one senior critical care doctor and one senior obstetric doctor. Likewise, these doctors also made decisions to discharge patients or to transfer patients to general wards. Patients were excluded if they had known hematologic diseases (including leukemia, thrombotic thrombocytopenic purpura, and other hematologic diseases) or history of recent blood transfusion (less than two weeks). According to the APACHE II scores criterion of Knaus WA, 1985,[20] the recorded value in our study was based on the most deranged reading during each patient's initial 24h in an ICU, so we excluded the patients who died or were discharged less than 24 hours.

For peer review only - http://bmjopen.bmj.com/site/about/guidelines.xhtml

Data collection

Demographic and clinical characteristics including age, gestational age, parity, pregnancy status at admission, diagnosis at entry and during ICU stay, and length of hospital stay were collected. The cesarean section was considered an emergency intervention. The pregnancy associated with cardiac disease include congenital and acquired heart disease during pregnancy. Diagnosis of acute kidney injury (AKI) was based on the Acute Kidney Injury Network (AKIN) criteria.[21] AKI was defined as an absolute increase in serum creatinine of more than or equal to 0.3 mg/dl, a percentage increase in serum creatinine of more than or equal to 50% (1.5 fold from baseline) within 48 hours, or reduction in urine output (documented oliguria of less than 0.5 ml/Kg per hour for more than six hours).[21] APACHE II scores were calculated using the worst value of 12 acute physiological variables within 24 hours of presentation. These variables included temperature, blood pressure, heart rate, respiratory rate, arterial oxygenation, arterial pH, serum sodium, serum potassium, serum creatinine, hematocrit, white blood cell count, and Glasgow Coma scores. If the patient was sedated at the time of ICU admission, the last Glasgow Coma Scale obtained prior to sedation was collected. RDW, hemoglobin level, hematocrit and mean corpuscular volume (MCV) of all patients included in this study were determined at admission using a Beckman Coulter LH-750 Hematology Analyzer (Beckman Coulter, Inc., Fullerton, California), as a part of the complete blood cell count. Normal reference range for RDW in the hospital laboratory is 10.9% to 15.4%. In addition, we got the number of birth (from January 1, 2008 to December 31, 2013) from hospital database in order to count ICU admission rate in the hospital.

For peer review only - http://bmjopen.bmj.com/site/about/guidelines.xhtml

Study outcomes

All patients were followed up during hospitalization. The primary end point of the study was hospital mortality. The primary predictor of interest was RDW measured at ICU admission.

Statistics

All continuous variables were presented as means ± standard deviations, or medians with interquartile ranges as appropriate. Categorical data were summarized as number or percentage. We divided RDW into tertiles, and compared the demographics, diagnosis, clinical characteristics, laboratory test results, and APACHE-II scores of patients using analysis of variance or Kruskal-Wallis tests for continuous variables, and Chi-square or Fisher's exact tests for categorical variables. Univariate logistic regression analysis was conducted to examine the association between mortality and each of the predictors, separately. Multivariate logistic regression was further utilized to determine independent predictors of ICU mortality after adjusting for potential confounding factors. An appealing receiver operating characteristic (ROC) curve was used to examine the performance of APACHE-II scores and RDW in predicting hospital mortality. The curve represented a sensitivity plot *versus* 1-specificity. The area under the curve (AUC) was derived from the ROC curve, and the Youden index was adopted to define the optimal cut-off value.[22] We also constructed an ROC curve for the combined APACHE-II score and RDW results for predicting hospital mortality according to the weighted sum formula derived from multivariate logistic regression: logit (mortality) = 0.162 x APACHE-II score + 0.203 x RDW - 7.503; wherein, logit

For peer review only - http://bmjopen.bmj.com/site/about/guidelines.xhtml

(mortality) is the logarithm of the odds of a critically ill obstetric patient dying in the ICU. Differences between the AUC were detected by Delong's testwhich was a nonparametric approach and could generate an estimated covariance matrix by using the theory on generalized U-statistics.[23] Two-sided *P*-values <0.05 were considered statistically significant, and all analyses were performed with R software (http://www.cran.r-project.org/).

Results

Population

A total of 20570 births was reported from January 1, 2008 to December 31, 2013 in Shandong Provincial Hospital, among which 447 obstetric patients were admitted to ICU. ICU admission rate was 21.73 per 1,000 births in the hospital. 8 patients who were diagnosed with hematologic disease and 59 patients who received red blood cell transfusion within two weeks were excluded. 2 patients who died within 24 hours after ICU admission were also excluded. 2 patients were excluded for missing data. Thus, in the final analysis, a total of 376 patients were included in this study.

Association between RDW and hospital mortality

A total of 20 deaths occurred in this cohort during the study period. In this study, heart failure was the major cause of death in the cohort (n=8; 40.0%), followed by acute fatty liver of pregnancy (n=5; 25%), postpartum hemorrhage (n=2), hemorrhagic shock caused by liver tumor rupture (n=1), HELLP syndrome (n=1), acute pulmonary embolism (n=1), stroke (n=1), and liver

For peer review only - http://bmjopen.bmj.com/site/about/guidelines.xhtml

For peer review only

failure caused by severe hepatitis B (n=1). The hospital mortality was 5.32%, and RDW ranged from 10.7% to 24.0% (median, 14.8%; interquartile range, 13.4% to 16.0%).

Participants were divided into 3 categories on the basis of their baseline tertiles of RDW level: tertile I, 10.7%-13.9%; tertile II, 13.9%-15.6%; tertile III, 15.6%-20.4% (all $P<0.001$). There was no difference in age, gestational weeks, primary reasons for ICU admission, AKI morbidity, as well as in the total length of stay in hospital (TLSH) among the 3 tertiles. Hb, MCV and HCT significantly decreased with the increase of RDW, while APACHE II scores and hospital mortality significantly increased with the increase of RDW (Table 1). A significant association was found between baseline RDW levels and hospital mortality (odds ratio per percent increase in RDW, 1.21; 95% CI, 1.02 to 1.57).

Regression analysis for hospital mortality

Univariate logistic regression analysis demonstrated that patients with higher RDW, higher APACHE-II scores, higher AKI morbidity, and longer TLSH days had significantly greater death hazards (Table 2). Multivariate logistic regression analysis revealed that RDW, AKI and APACHE-II scores were independent predictors of hospital mortality (Table 3). The association of RDW and hospital mortality remained significant after adjusting for age, hemoglobin, MCV, hematocrit, APACHE-II score and AKI. RDW was a significant outcome predictor, which is independent of APACHE-II scores.

A ROC curve was drawn to evaluate the value for RDW and APACHE-II scores in predicting mortality (Figure 1). The AUC was calculated as 0.766

For peer review only - http://bmjopen.bmj.com/site/about/guidelines.xhtml

(95% CI, 0.705 to 0.826) for the APACHE-II score and 0.752 (95% CI, 0.684 to 0.862) for RDW. Optimal cut-off value of the APACHE-II score for predicting mortality was 5 points, which gave a sensitivity of 90.9% and a specificity of 60.7%. The optimal cut-off value of RDW was 16.1%, which gave a sensitivity of 68.2% and specificity of 77.9%. To further clarify whether RDW had an additive power with the APACHE-II score for hospital mortality, we combined RDW and the APACHE-II score to draw a third ROC curve, as shown in Figure 1. Compared with the APACHE-II score, adding RDW to APACHE-II scores improved the AUC from 0.766 to 0.872 (P<0.001). These results suggested that combining RDW and APACHE-II added to the ability to discriminate mortality risk. As in multivariate logistic regression analysis, AKI was another strong predictor for hospital mortality. We conducted the ROC curve analysis using all three variables including RDW, APACHE-II and AKI. Adding AKI improved the AUC from 0.872 to 0.884 (P>0.05), however, no significant difference was observed between the AUC derived from all three variables or that derived only from RDW and APACHE-II. The Delong's Z statistic and P value were equal to -0.668 and 0.504 respectively.

Discussion

The main finding of our study was that RDW was independently associated with hospital mortality in obstetric critical care patients . The association remained significant even after adjusting for APACHE-II scores, hemoglobin levels, hematocrit and mean corpuscular volume. Combining RDW and the APACHE-II score improved the prognostic accuracy of hospital mortality as predicted by APACHE-II alone in the study population.

For peer review only - http://bmjopen.bmj.com/site/about/guidelines.xhtml

We found that RDW was independently associated with hospital mortality in a Chinese obstetric ICU population. Patients were excluded if they had a history of recent blood transfusions because RDW could be increased in anemia or after blood transfusions.[11, 24] In this present study, higher level of RDW was associated with hospital mortality even after adjusting for hemoglobin and hematocrit. In addition, the independent association between RDW and hospital mortality was not eliminated even after adjusting for other potential confounders, including mean corpuscular volume, hemoglobin, hematocrit, APACHE-II score, gestational age and AKI. Our results were consistent with two previous population studies on critically ill patients.[25, 26] In addition, our results were also in line with the findings obtained in a single-center observational study in 2011[16], which showed independent association between RDW and all-cause mortality in critically ill patients.

Our unique findings indicated that combining RDW and APACHE-II score performed better than APACHE-II score alone in predicting hospital mortality in critically ill obstetric patients. The APACHE-II scoring system developed in 1985 has shown a positive correlation with hospital mortality, and was one of the most common models used for evaluating the severity of a disease in critically ill patients.[20] Previous studies[27-29] suggested APACHE II score was a good discriminator of illness severity in obstetric cohorts. The sensitivity and specificity of the APACHE II score was evaluated with the use of receiver operating characteristic curve analysis. In accordance with previous studies[27-29], the APACHE-II score was also demonstrated to have a good ability to predict hospital mortality (AUC=0.766) in the present study. Despite its moderate discriminative power alone (AUC=0.752), adding RDW to the

For peer review only - http://bmjopen.bmj.com/site/about/guidelines.xhtml

APACHE-II score resulted to a better increase in the AUC from 0.766 to 0.872 (*P*<0.001). Combining RDW with APACHE-II score significantly improved prognostic performance in critically ill obstetric patients. Similarly, another study by Wang *et al.*[16]also revealed that adding RDW to APACHE-II score significantly improved prognostic reliability of APACHE-II score in identifying critically ill patients. Since RDW is a simple, inexpensive, and widely available test as part of the complete blood count, these data may have significant clinical implications for determining prognosis in critically ill obstetric patients. However, no significant improvement on prognostic performance was observed when AKI was associated with RDW and APACHE-II score, even though AKI was another strong predictor for hospital mortality in multivariate logistic regression analysis.

The pathophysiologic mechanism underlying the association of higher RDW with worse outcomes in critically ill obstetric patients remains unclear. Generally, the increase in RDW reflects either impaired erythropoiesis, abnormal red blood cell survival, or both. Metabolic abnormalities such as shortening of telomere length, poor nutritional status,[30, 31] inflammation, [32-34] oxidative stress, [35, 36] dyslipidemia, hypertension, erythrocyte fragmentation, or alteration of erythropoietin function might contribute to RDW increase.[37] In normal parturition, the increase of RDW might be related to stimulus-induced reticulocytosis in the last few weeks.[29] This might not be the case in our study; wherein, even if five patients with spontaneous onset of labor were excluded, RDW remained as a significant predictor of mortality.

For peer review only - http://bmjopen.bmj.com/site/about/guidelines.xhtml

In this present study, the admission rate of 21.73 per 1,000 births was relatively high,[38-39] and the mean APACHE-II score was relatively low compared with other studies.[7, 16, 40] The lack of a high dependency unit as a referral center in local areas for complicated pregnancies could partially explain these difference.

This study had several limitations. First, we did not evaluatethe evolution of RDW, and thus, it remained unclear whether changes in RDW over time may provide additional prognostic information.Second, we only used the APACHE-II score to predict mortality in obstetric patients, which was limited by spontaneous improvement after delivery in peripartum women,[41-47] and inconsistent results.[48, 49] Finally, our study was a single-center study. The study was very heterogenous and the case number was relatively small, in future, a large population based study may validate and strengthen our findings.

In summary, the single-center study in China demonstrated that RDW was an independent predictor for mortality in obstetric critical care patients. Combining RDW and the APACHE-II score improved prognostic accuracy compared with the APACHE-II score model alone.

Acknowledgements

This study was funded by the National Natural Science Foundation of China (NSFC 81200238), the Research Foundation for Excellent Young and Middle-aged Scientists of Shandong Province (BS2011YY043, BS2011YY052),the Young Scholars Program of Shandong University (2016WLJH23), the Clinical medicine science and technology innovation plan of Jinan Bureau of Science

For peer review only - http://bmjopen.bmj.com/site/about/guidelines.xhtml

& Technology(201602164), the Shandong Provincial Department of Science & Techno-logy research and development plan (2016GSF201052),and the Natural Science Foundation of Shandong Province of China (ZR2013HM062).

Conflict of Interests

The authors declare that there is no conflict of interests regarding the publication of this paper.

References

1. World Health Organization. World Health Statistics 2014. Geneva, World Health Organization; 2014.

2. Zeeman GG. Obstetric critical care: A blueprint for improved outcomes. Crit Care Med 2006; 34:S208–S214.

3. Soubra SH, Guntupalli KK. Critical illness in pregnancy: an overview. Crit Care Med 2005;33(10 suppl):S248-S255.

4. Scarpinato L, Gerber D. Critically ill obstetrical patients: outcome and predictability. Crit Care Med 1995;23(8):1449-1451.

5. Gopalan PD, Muckart DJ. The critically ill obstetric patient: what's the score? Int J ObstetAnesth 2004;13(3):144-145.

6. Ramsay MM, James D, Steer P, et al, editors. Normal values in pregnancy. London: W.B. Saunders; 2000.

For peer review only

7. Lapinsky SE, Hallett D, Collop N, et al. Evaluation of standard and modified severity of illness scores in the obstetric patient.J Crit Care 2011 Oct;26(5):535.e1-7.

8. Harrison D, Penny J, Yentis S, et al. Case mix, outcome and activity for obstetric admissions to adult, general critical care units: A secondary analysis of the ICNARC Case Mix Programme Database. Crit Care 2005; 9(Suppl 3):S25–S37.

9. Paternina-Caicedo A, Rojas-Suarez J, Duenas-Castel C, et al. Mortality risk prediction with an updated Acute Physiology and Chronic Health Evaluation II Score in critically ill obstetric patients: A cohort study. J Intensive Care Med 2015 Feb;30(2):97-102.

10. Perkins SL. Examination of blood and bone marrow. In: Greer JP, Foerster J, Lukens JN, Rodgers GM, Paraksevas F, Glader BE, eds. Wintrobe's Clinical Hematology. 11th ed. Salt Lake City, Utah: Lippincott Wilkins & Williams; 2003:5–25.

11. McKenzie SD. Introduction to anemia. In: McKenzie SD, ed. Clinical Laboratory Hematology. Saddle River, NJ: Pearson Prentice-Hall; 2003:161–188.

12. Tonelli M, Sacks F, Arnold M, et al. For the Cholesterol and Recurrent Events (CARE) Trial InvestigatorsCirculation.Relation Between Red Blood Cell Distribution Width and Cardiovascular Event Rate in People With Coronary Disease. Circulation 2008;117:163–8.

For peer review only - http://bmjopen.bmj.com/site/about/guidelines.xhtml

13. Felker GM, Allen LA, Pocock S, et al. CHARM Investigators: Red cell distribution width as a novel prognostic marker in heart failure: data from the CHARM Program and the Duke Databank for Cardiovascular Diseases. J Am CollCardiol 2007;50:40-47.

14. Förhécz Z, Gombos T, Borgulya G, et al. Red cell distribution width in heart failure: prediction of clinical events and relationship with markers of ineffective erythropoiesis, inflammation, renal function, and nutritional state. Am Heart J 2009; 158:659–66.

15. Pascual-Figal DA, Bonaque JC, Redondo B, et al. Red blood cell distribution width predicts long-term outcome regardless of anaemia status in acute heart failure patients. Eur J Heart Fail 2009;11:840–6.

16. Wang F, Pan W, Pan S, et al. Red cell distribution width as a novel predictor of mortality in ICU patients.Ann Med 2011 Feb;43(1):40-6.

17. Ani C, Ovbiagele B. Elevated red blood cell distribution width predicts mortality in persons with known stroke. J NeurolSci 2009;277:103–8.

18. Purtle SW, Moromizato T, McKane CK, et al. The association of red cell distribution width at hospital discharge and out-of-hospital mortality following critical illness.Crit Care Med 2014 Apr;42(4):918-29.

19. Kim CH, Park JT, Kim EJ, et al. An increase in red blood celldistributionwidth from baseline predicts mortality in patients with severe sepsis or septic shock.Crit Care 2013 Dec 9;17(6):R282.

20. Youden WJ: Index for rating diagnostic tests. Cancer 1950;3:32–5.

For peer review only - http://bmjopen.bmj.com/site/about/guidelines.xhtml

21. DeLong ER, DeLong DM, Clarke-Pearson DL. Comparing the areasundertwo or morecorrelatedreceiver operating characteristiccurves: a nonparametricapproach.Biometrics 1988 Sep;44(3):837-45.

22. Weiss G, Goodnough LT. Anemia of chronic disease. N Engl J Med 2005;352:1011–1023.

23. Sabina Hunziker, Leo A Celi, Joon Lee, et al. Red cell distribution width improves the simplified acute physiology score for risk prediction in unselected critically ill patients. Critical Care 2012,16:R89.

24. Heidi S. Bazick, Domingo Chang, KarthikMahadevappa, et al. Red cell distribution width and all-cause mortality in critically ill patients. Crit Care Med 2011;39:1913–1921.

25. Knaus WA, Draper EA, Wagner DP, et al. APACHE II: a severity of disease classification system. Crit Care Med 1985;13:818–29.

26. Knaus WA, Draper EA, Wagner DP, et al. APACHE II: a severity of disease classification system. Crit Care Med 1985;13:818–29.

27. Hazelgrove JF, Price C, Pappachan VJ, et al. Multicenter study of obstetric admissions to 14 intensive care units in southern England.Crit Care Med 2001 Apr;29(4):770-5.

28. Harrison D, Penny JA, Yentis SM, et al. Case mix, outcome and activity for obstetric admissions to adult, general critical care units: a secondary analysis of the ICNARC case mix programme database. Critical Care 2005;9(suppl 3):S25-S37.

For peer review only

For peer review only - http://bmjopen.bmj.com/site/about/guidelines.xhtml

29. Arabi Y, Goraj R, Horsfall D. Scoring systems in obstetric patients requiring intensive care in Saudi Arabia: a ten year review. Chest 1999;116:286S.

30. Duffy TP. Approach to the patient with anemia. In: Kelley WN, Dupont HL, Glick JH, Harris ED, Hathaway DR, Hazzard WR, Holmes EW, Hudson LD, Humes HD, Paty DW, eds. Kelley's Textbook of Internal Medicine. 3rd ed. Philadelphia, Pa: Lippincott Williams and Wilkins; 1997:1295–1299.

31. Forhecz Z, Gombos T, Borgulya G, et al. Red cell distribution width in heart failure: prediction of clinical events and relationship with markers of ineffective erythropoiesis, inflammation, renal function, and nutritional state. Am Heart J 2009, 158:659-666.

32. Ridker PM, Rifai N, Clearfield M, et al. Measurement of C-reactive protein for the targeting of statin therapy in the primary prevention of acute coronary events. N Engl J Med 2001;344:1959 –1965.

33. Lippi G, Targher G, Montagnana M, et al. Relation between red blood cell distribution width and inflammatory biomarkers in a large cohort of unselected outpatients. Arch Pathol Lab Med 2009;133:628–632.

34. Sesso HD, Buring JE, Rifai N, et al. C-reactive protein and the risk of developing hypertension. JAMA 2003;290:2945–2951.

35. Kolls JK. Oxidative stress in sepsis: A redox redux. J Clin Invest 2006;116:860–863.

For peer review only

For peer review only - http://bmjopen.bmj.com/site/about/guidelines.xhtml

36. Ghaffari S. Oxidative stress in the regulation of normal and neoplastic hematopoiesis. Antioxid Redox Signal 2008;10:1923–1940.

37. Salvagno GL, Sanchis-Gomar F,Picanza A.Lippi G. Red blood cell distribution width: A simple parameter with multiple clinical applications. Crit Rev Clin Lab Sci.2014 Dec 23:1-20.

38. Rojas-Suarez J, Paternina-Caicedo AJ, Miranda J, et al. Comparison of severity-of-illness scores in critically ill obstetric patients: a 6-year retrospective cohort. Crit Care Med 2014;42:1047–1054.

39. Hazelgrove JF, Price C, Pappachan VJ, et al. Multicenter study of obstetric admissions to 14 intensive care units in southern England.Crit Care Med 2001 Apr;29(4):770-5.

40. Karnad DR, Lapsia V, Krishnan A, et al. Prognostic factors in obstetric patients admitted to an Indian intensive care unit.Crit Care Med 2004 Jun;32(6):1294-9.

41. Higgins TL, Teres D, Copes W. Updated Mortality Probability Model (MPM-III). Chest 2005;128(4 Suppl):348S.

42. Knaus WA, Draper EA, Wagner DP, et al.APACHE II: A severity of disease classification system. Crit Care Med 1985;13:818–829.

43. Le Gall JR, Lemeshow S, Saulnier F. A new Simplified Acute Physiology Score (SAPS II) based on a European/North American multicenter study. JAMA 1993;270:2957–2963.

For peer review only

For peer review only - http://bmjopen.bmj.com/site/about/guidelines.xhtml

44. Lemeshow S, Teres D, Klar J, et al. Mortality Probability Models (MPM II) based on an international cohort of intensive care unit patients. JAMA 1993;270:2478–2486.

45. Metnitz PG, Moreno RP, Almeida E,et al. SAPS 3 Investigators: SAPS 3–From evaluation of the patient to evaluation of the intensive care unit. Part 1: Objectives, methods and cohort description. Intensive Care Med 2005;31:1336–1344.

46. Moreno RP, Metnitz PG, Almeida E, et al. SAPS 3 Investigators: SAPS 3–From evaluation of the patient to evaluation of the intensive care unit. Part 2: Development of a prognostic model for hospital mortality at ICU admission. Intensive Care Med 2005;31: 1345–1355.

47. Higgins TL, Kramer AA, Nathanson BH, et al. Prospective validation of the intensive care unit admission Mortality Probability Model (MPM0-III). Crit Care Med 2009;37:1619–1623.

48. Stevens TA, Carroll MA, Promecene PA, et al. Utility of acute physiology, age, and chronic health evaluation (APACHE III) score in maternal admissions to the intensive care unit. Am J ObstetGynecol 2006;194(5):e13-e15.

49. el-Solh AA, Grant BJ. A comparison of severity of illness scoring systems for critically ill obstetric patients. Chest 1996;110(5): 1299-1304.

For peer review only

For peer review only - http://bmjopen.bmj.com/site/about/guidelines.xhtml

Table 1. Baseline clinical and laboratory characteristics by tertile of RDW at critical care initiation

	Tertile I	Tertile II	Tertile III	*P*-value
**RDW (%)**	10.7-13.9	13.9-15.6	15.6-20.4	<0.001
N	131	126	119	-
**Age (years)**	29.6 ± 4.3	29.3 ± 5.6	29.6 ± 5.9	0.84
**Gestational age (weeks)**	35 (32-37)	36 (33-39)	36 (32-38)	0.327
**Primary diagnosis to ICU admission (*n*, %)**				
Hypertensive disorder of pregnancy	50(38.17%)	55(43.65%)	52(43.70%)	0.417
HELLP Syndrome	12(9.16%)	10(7.94%)	8(6.72%)	0.322
Acute fatty liver of pregnancy	10(7.63%)	8(6.35%)	7(5.88%)	0.485
Obstetric sepsis	5(3.82%)	4(3.17%)	3(2.52%)	0.657
Cardiovascular disease	42 (32.06%)	31 (24.60%)	26 (21.85%)	0.162
Gastrointestinal disease	2 (1.53%)	5 (3.97%)	4 (3.36%)	0.476
Stroke	2 (1.53%)	3 (2.38%)	0 (0)	0.332
Pulmonary embolism	1 (0.76%)	0 (0)	1 (0.84%)	0.766
Others	7(5.34%)	10(7.94%)	18(15.13)	0.142
**Hemoglobin (g/L)**	109.5 ± 19.7	100.1 ± 20.7	92.5 ± 24.1	<0.001
**MCV(fL)**	89.8 ± 5.1	86.7 ± 6.0	83.6 ± 11.4	<0.001
**HCT (%)**	32.6 ± 5.8	30.5 ± 6.3	28.8 ± 6.9	<0.001
**APACHE II score (points)**	2 (2-6)	5 (3-6)	10 (6-22)	0.017
**AKI (*n*, %)**	14 (10.69%)	17 (13.49%)	14 (11.76%)	0.207
**TLSH (days)**	8 (7-12)	8 (6-13)	8 (7-12)	0.203
**Hospital Mortality (*n*, %)**	0 (0%)	5 (3.97%)	15 (12.61%)	<0.001

For peer review only

For peer review only - http://bmjopen.bmj.com/site/about/guidelines.xhtml

Note: RDW, red cell distribution width; MCV,mean corpuscular volume; HCT, hematocrit; APACHE-II score, Acute Physiology and Chronic Health Evaluation II score; AKI, acute kidney injury; TLSH, total length of stay in hospital. Values are presented as mean ± standard deviations or number (percentage).

For peer review only

For peer review only - http://bmjopen.bmj.com/site/about/guidelines.xhtml

Table 2. Univariate odds ratios of variables for predicting mortality

Variables	Odds ratio	95% CI	*P*-value
Age (years)	1.048	0.969-1.133	0.244
Hemoglobin (g/L)	0.997	0.977-1.017	0.763
MCV (fL)	0.962	0.919-1.006	0.098
Hematocrit (%)	0.997	0.934-1.064	0.929
APACHE-II score (points)	1.192	1.124-1.265	< 0.001
AKI (%)	16.61	6.580-42.014	< 0.001
TLSH (days)	0.803	0.691-0.933	0.004
gestational age (weeks)	1.023	0.920-1.138	0.677
RDW (%)	1.309	1.150-1.489	<0.001

Note: CI, confidence interval; RDW, red cell distribution width; MCV, mean corpuscular volume; HCT, hematocrit; APACHE-II score, Acute Physiology and Chronic Health Evaluation II score; AKI, acute kidney injury; TLSH, total length of stay in hospital.

For peer review only - http://bmjopen.bmj.com/site/about/guidelines.xhtml

Table 3. Independent predictors of mortality by multivariate logistic regression analysis

Variables	Odds ratio	95% CI	*P* value
APACHE-II (points)	1.189	1.071-1.319	0.001
AKI (%)	23.784	6.129-92.296	<0.001
RDW (%)	1.401	1.156-1.697	0.001

Note: Independent variables include age, hemoglobin, MCV, hematocrit, APACHE-II score, AKI, TLSH, gestational age, and RDW. Due to the high correlation between hemoglobin and hematocrit, hemoglobin was first regressed on hematocrit; and placed the residual and hematocrit in the multivariate regression. RDW, red cell distribution width; MCV, mean corpuscular volume; HCT, hematocrit; APACHE-II score, Acute Physiology and Chronic Health Evaluation II score; AKI, acute kidney injury; TLSH, total length of stay in hospital.

For peer review only - http://bmjopen.bmj.com/site/about/guidelines.xhtml

473 Figure legend:

474 Figure 1: ROC curve for APACHE II score, RDW, and the combination of both
475 in predicting hospital mortality.

For peer review only

For peer review only - http://bmjopen.bmj.com/site/about/guidelines.xhtml

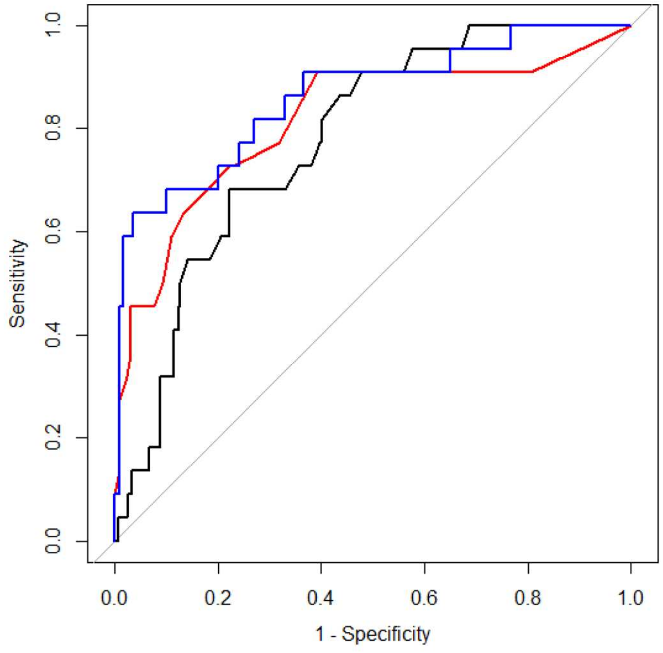

Figure 1

ROC curve for APACHE II score, RDW, and the combination of both in predicting hospital mortality. Compared with the APACHE-II score, adding RDW to APACHE-II scores improved the AUC from 0.766 to 0.872 (*P*<0.001). — RDW (AUC 0.752 ± 0.062); — APACHE-II score (AUC 0.766 ± 0.031); — RDW+ APACHE-II score (AUC 0.872 ± 0.055); ROC, receiver operating characteristic; AUC, area under the curve; APACHE-II score, Acute Physiology and Chronic Health Evaluation II score; RDW, red cell distribution width.

173x177mm (300 x 300 DPI)

Red blood cell distribution width as a risk factor for in-hospital mortality in obstetric patients admitted to Intensive care unit – a single center, retrospective cohort study.

Journal:	*BMJ Open*
Manuscript ID	bmjopen-2016-012849.R3
Article Type:	Research
Date Submitted by the Author:	30-Mar-2017
Complete List of Authors:	Chu, Yufeng; Shandong Provincial Hospital Affiliated to Shandong University<br>Yuan, Zhongshang; Shandong University, Department of Epidemiology and Biostatistics<br>Meng, Mei; Shandong Provincial Hospital Affiliated to Shandong University<br>Zhou, Haiyan; Shandong Tumor Hospital and Institute<br>Wang, Chunting; Shandong Provincial Hospital Affiliated to Shandong University<br>Yang, Gong; Vanderbilt School of Medicine, Department of Medicine<br>Ren, Hongsheng; Shandong Provincial Hospital Affiliated to Shandong University, Department of Intensive Care Unit
**Primary Subject Heading**:	Intensive care
Secondary Subject Heading:	Obstetrics and gynaecology
Keywords:	APACHE-II score, Critical care, Mortality, OBSTETRICS, Red cell distribution width

SCHOLARONE™
Manuscripts

TITLE PAGE

Red blood cell distribution width as a risk factor for in-hospital mortality in obstetric patients admitted to Intensive care unit – a single center, retrospective cohort study.

Yufeng Chu,[a,*] Zhongshang Yuan,[b,*] Mei Meng,[a] Haiyan Zhou,[c] Chunting Wang,[a] Gong yang[d], Hongsheng Ren[a]

[a] *Department of Intensive Care Unit, Shandong Provincial Hospital Affiliated to Shandong University, Shandong University, Jinan, P.R. China*

[b] *Department of Epidemiology and Biostatistics, School of Public Health, Shandong University, Jinan, P.R. China*

[c] *Department of Medical Oncology ,Shandong Tumor Hospital, Jinan, P.R. China*

[d] *Vanderbilt Epidemiology Center, Department of Medicine, Vanderbilt University Medical Center, Nashville, TN, USA*

Yufeng Chu and Zhongshang Yuan are co-first authors.

Corresponding author:

Hongsheng Ren, MD, Ph. D.

Department of Intensive Care Unit

Shandong Provincial Hospital Affiliated to Shandong University

No. 324 Jingwu Road, Jinan, Shandong, 250012, P.R. China

Email: sdslicu@163.com

For peer review only

For peer review only - http://bmjopen.bmj.com/site/about/guidelines.xhtml

Abstract

Background: Red blood cell distribution width (RDW) has been shown to predict mortality in critically ill patients. To our knowledge, it has not been evaluated whether RDW is associated with clinical outcomes of obstetric patients requiring critical care.

Methods: This is a single-center, retrospective observational study of obstetric patients admitted to intensive care unit (ICU). Patients were excluded from the analysis if they had known hematologic diseases or recently underwent blood transfusion. Patients who died or were discharged from ICU within 24 hours of admission were also excluded. Patient clinical characteristics at ICU admission were retrieved from medical chart. Multiple logistic regression was used to estimate odds ratios (OR) and 95% confidence intervals (95% CI) for in-hospital mortality associated with RDW. The receiver operating characteristic curve was used to examine the performance of RDW, alone or in combination with Acute Physiology and Chronic Health Evaluation II score (APACHE II), in predicting in-hospital mortality.

Results: A total of 376 patients were included in this study. The hospital mortality was 5.32%. A significant association was found between baseline RDW levels and hospital mortality (odds ratio per percent increase in RDW, 1.21; 95% CI, 1.02 to 1.57). Further adjustment for hematocrit and other potential confounders did not appreciably alter the result (P<0.001).The area under the curve (AUC) for in-hospital mortality by RDW was similar to that by

For peer review only - http://bmjopen.bmj.com/site/about/guidelines.xhtml

APACHE II score (0.752 vs 0.766). Combination of these two factors resulted in substantial improvement in risk prediction, with AUC of 0.872 (P<0.001).

Conclusions: This study suggests that RDW is an independent predictor for in-hospital mortality among ICU-admitted obstetric patients. Combining RDW and APACHE II scores could significantly improve in-hospital prognostic prediction among these critically-ill obstetric patients.

Key words: APACHE-II score; Critical care; Mortality; Obstetrics; Red cell distribution width

Strengths and limitations of this study

• To our knowledge, this is the first report of red cell distribution width (RDW) as an independent prognostic predictor of clinical outcomes in obstetric critical care patients.

• The study finding suggests that RDW, a routinely measured clinical laboratory test with high reproducibility, may have direct clinical implications and may aid the improvement of critical care for obstetric patients.

• This is a single-center study. Replication in other populations/settings is warranted.

For peer review only

Introduction

Maternal mortality ratio (MMR) remains high, despite the advances in the critical care of obstetric patients, especially in developing countries.[1, 2] The use of scoring systems to assess its severity and predict mortality may help identify obstetric patients who truly require intensive care.[3] Acute Physiology and Chronic Health Evaluation II (APACHE II) score is a predictive score for mortality that are widely used in ICUs. However, results from obstetric patients requiring critical care have been mixed. Some studies suggest this is a good predictor for illness severity, but other more recent studies have shown that this score has overestimated mortality risk.[4, 5, 6, 7-9] Therefore, there is a real need to identify new factors in order to improve the assessment of illness severity and prediction of clinical outcomes for critically-ill obstetric patients.

Red cell distribution width (RDW), a routinely measured clinical laboratory test with high reproducibility, reflects the degree of heterogeneity of erythrocyte volume.[10] RDW has been used to differentiate anemia types over the past decades.[11] Recently, RDW has been shown to be a novel, independent prognostic marker for mortality, mainly in patients with cardiovascular disease and strokes, as well as in critically ill patients.[12-19] However, to our knowledge, no study has directly examined the prognostic performance of RDW among obstetric patients requiring critical care.

In the study, we conducted a retrospective cohort study of ICU-admitted obstetric patients to evaluate whether RDW at ICU admission was associated with in-hospital mortality and examined the performance of RDW, alone or in

For peer review only

combination with Acute Physiology and Chronic Health Evaluation II score (APACHE II), in predicting risk for in-hospital mortality.

Methods

The study was carried out at the Shandong Provincial Hospital Affiliated to Shandong University, Jinan, China. The hospital is a 1500-bed tertiary academic hospital with 20 ICU beds, which provides primary as well as tertiary care to an ethnically and socioeconomically diverse population within Shandong province and the surrounding region. The study was approved by the Institutional Review Board of the hospital. Obstetric patients consecutively admitted in the ICU for at least 24 hours from January 1, 2008 to December 31, 2013 were included in this retrospective observational study. The requirement for patient consent was waived given the retrospective nature of this study design, i.e., no direct/indirect patient care intervention, and that all identifiable information was removed. Obstetric patients were defined as pregnant women or up to six weeks postpartum. The decision to transfer patients into the ICU was made by at least one senior critical care doctor and one senior obstetric doctor. Likewise, these doctors also made decisions to discharge patients or to transfer patients to general wards. Patients were excluded if they had known hematologic diseases (including leukemia, thrombotic thrombocytopenic purpura, and other hematologic diseases) or history of recent blood transfusion (less than two weeks). According to the APACHE II score criterion of Knaus WA, 1985,[20] the recorded value in our study was based on the most deranged reading during each patient's initial

For peer review only - http://bmjopen.bmj.com/site/about/guidelines.xhtml

24h in an ICU, so patients who died or were discharged less than 24 hours were excluded from the study.

Data collection

Demographic and clinical characteristics including age, gestational age, parity, pregnancy status at admission, diagnosis at entry and during ICU stay, and length of hospital stay were all collected. Cesarean section was considered an emergency intervention. The pregnancy associated with cardiac disease include congenital and acquired heart disease during pregnancy. Diagnosis of acute kidney injury (AKI) was based on the Acute Kidney Injury Network (AKIN) criteria.[21] AKI was defined as an absolute increase in serum creatinine of more than or equal to 0.3 mg/dl, a percentage increase in serum creatinine of more than or equal to 50% (1.5 fold from baseline) within 48 hours, or reduction in urine output (documented oliguria of less than 0.5 ml/Kg per hour for more than six hours).[21] APACHE II scores were calculated using the worst value of 12 acute physiological variables within 24 hours of presentation. These variables included temperature, blood pressure, heart rate, respiratory rate, arterial oxygenation, arterial pH, serum sodium, serum potassium, serum creatinine, hematocrit, white blood cell count, and Glasgow Coma scores. If the patient was sedated at the time of ICU admission, the last Glasgow Coma Scale obtained prior to sedation was collected. RDW, hemoglobin level, hematocrit and mean corpuscular volume (MCV) of all patients included in this study were determined at admission using a Beckman Coulter LH-750 Hematology Analyzer (Beckman Coulter, Inc., Fullerton, California), as a part of the complete blood cell count. Normal reference range for RDW in the

For peer review only

For peer review only - http://bmjopen.bmj.com/site/about/guidelines.xhtml

hospital laboratory is between 10.9% and 15.4%. In addition, we obtained the number of birth (from January 1, 2008 to December 31, 2013) from hospital database in order to calculate ICU admission rate in the hospital.

Study outcomes

All patients were followed up during hospitalization. The primary end point of the study was in-hospital mortality. The primary predictor of interest was RDW measured at ICU admission.

Statistics

All continuous variables were presented as means ± standard deviations, or medians with interquartile ranges as appropriate. Categorical data were summarized as number or percentage. Patient characteristics across tertiles of RDW were compared using analysis of variance or Kruskal-Wallis tests for continuous variables and Chi-square or Fisher's exact tests for categorical variables. Logistic regression analysis was conducted to estimate odds ratios (OR) and 95% confidence intervals (95% CI) for in-hospital mortality associated with RDW and other clinical parameters after adjustment for potential confounding factors. The receiver operating characteristic (ROC) curve was used to examine the performance of RDW, alone or in combination with other clinical parameters such as APACHE II score, in predicting in-hospital mortality. The curve represented a sensitivity plot *versus* 1-specificity. The area under the curve (AUC) was derived from the ROC curve, and the Youden index was adopted to define the optimal cut-off value.[22] We also constructed an ROC curve for the combined APACHE-II score and RDW results for predicting in-hospital mortality according to the weighted sum

For peer review only - http://bmjopen.bmj.com/site/about/guidelines.xhtml

formula derived from multivariate logistic regression: logit (mortality) = 0.162 x APACHE-II score + 0.203 x RDW - 7.503; wherein, logit (mortality) is the logarithm of the odds of a critically ill obstetric patient dying in the ICU. Differences between the AUC were detected by Delong's test, which was a nonparametric approach and could generate an estimated covariance matrix by using the theory on generalized U-statistics.[23] Two-sided *P*-values <0.05 were considered statistically significant. All analyses were performed with R software (http://www.cran.r-project.org/).

Results

Population

A total of 20570 births were reported from January 1, 2008 to December 31, 2013 in Shandong Provincial Hospital, among which 447 obstetric patients were admitted to the ICU. ICU admission rate was 21.73 per 1,000 births in the hospital. 8 patients who were diagnosed with hematologic disease and 59 patients who received red blood cell transfusion within two weeks were excluded. 2 patients who died within 24 hours after ICU admission were also excluded. Also 2 patients were excluded for missing data. Thus, in the final analysis, a total of 376 patients were included in this study.

Association between RDW and hospital mortality

A total of 20 deaths occurred in this cohort during the study period. In this study, heart failure was the major cause of death in the cohort (n=8; 40.0%), followed by acute fatty liver of pregnancy (n=5; 25%), postpartum hemorrhage

For peer review only - http://bmjopen.bmj.com/site/about/guidelines.xhtml

(n=2; 10%), hemorrhagic shock caused by liver tumor rupture (n=1; 5%), HELLP syndrome (n=1; 5%), acute pulmonary embolism (n=1; 5%), stroke (n=1; 5%), and liver failure caused by severe hepatitis B (n=1; 5%). The in-hospital mortality was 5.32%.

RDW ranged from 10.7% to 24.0% (median, 14.8%; interquartile range, 13.4% to 16.0%). Table 1 shows the comparison of patient clinical characteristics across tertiles of RDW. RDW levels were inversely associated with levels of Hb, MCV and HCT, but positively associated with APACHE II score and in-hospital mortality. No difference was found in distributions of age, gestational weeks, primary reasons for ICU admission, AKI morbidity, or the total length of stay in hospital (TLSH) across RDW tertiles.

Regression analysis for in-hospital mortality

Univariate logistic regression analysis demonstrated that patients with higher RDW, higher APACHE-II scores, higher AKI morbidity, and longer TLSH days had significantly greater death hazards (Table 2). Per percent increase in RDW was associated with 21% increase in the risk, with OR of 1.21 (95% CI, 1.02 to 1.57).

Multivariate logistic regression analysis revealed that RDW, AKI and APACHE-II scores were independent predictors of in-hospital mortality (Table 3). The association of RDW and in-hospital mortality remained significant after adjusting for age, hemoglobin, MCV, hematocrit, APACHE-II score and AKI. RDW was a significant outcome predictor, which is independent of APACHE-II scores.

For peer review only - http://bmjopen.bmj.com/site/about/guidelines.xhtml

A ROC curve was drawn to evaluate the value for RDW and APACHE-II scores in predicting mortality (Figure 1). RDW and APACHE-II score were equally sensitive in prognostic prediction, with AUC of 0.766 (95% CI, 0.705 to 0.826) for the APACHE-II score and 0.752 (95% CI, 0.684 to 0.862) for RDW. Optimal cut-off value of the APACHE-II score for predicting mortality was 5 points, which yielded sensitivity and specificity of 90.9% and 60.7%. The optimal cut-off value of RDW was 16.1%, which resulted in sensitivity and specificity of 68.2% and 77.9%, respectively. We further combined RDW and the APACHE-II score to draw a third ROC curve, as shown in Figure 1, yielding much greater discriminatory capacity for in-hospital mortality, with AUC of 0.872. As shown in the multiple logistic regression analysis (Table 2), AKI was also significantly associated with in-hospital mortality. However, no appreciable improvement of prognostic performance was observed when further incorporating AKI into the prediction model. AUC derived from all three variables was 0.884.The Delong's *Z* statistic was -0.668 and *P* value was 0.504.

Discussion

The main finding of our study was that RDW was independently associated with in-hospital mortality in obstetric critical care patients . The association remained significant after adjusting for APACHE-II scores, hemoglobin levels, hematocrit and mean corpuscular volume. revealed the first evidence that the prognostic performance of RDW for in-hospital mortality among ICU-admitted obstetric patients was similar to APACHE-II score, a widely-accepted

For peer review only - http://bmjopen.bmj.com/site/about/guidelines.xhtml

predictor of clinical outcomes in critically ill patients. Combination of RDW and APACHE-II score provided even greater predictive power than either alone.

RDW has been associated with all-cause mortality in critically ill patients.[16,24,25] We for the first time specifically evaluated the association among obstetric patients requiring critical care. We found that RDW was independently associated with in-hospital mortality inthese patients. Patients were excluded if they had a history of recent blood transfusions because RDW could be increased in anemia or after blood transfusions.[11, 26] In this present study, higher level of RDW was associated with in-hospital mortality even after adjusting for hemoglobin and hematocrit. In addition, the independent association between RDW and hospital mortality was not eliminated after further adjusting for other potential confounders such as mean corpuscular volume, hemoglobin, hematocrit, APACHE-II score, gestational age and AKI. Our findings indicated that combining RDW and APACHE-II score performed better than APACHE-II score alone in predicting in-hospital mortality in critically ill obstetric patients.

The APACHE-II scoring system developed in 1985 has shown a positive correlation with hospital mortality, and was one of the most common models used for evaluating the severity of a disease in critically ill patients.[20] The sensitivity and specificity of the APACHE II score was evaluated with the use of receiver operating characteristic curve analysis in the present study. In accordance with previous studies[27-29], the APACHE-II score was demonstrated to have a moderate discriminative ability to predict in-hospital mortality (AUC=0.766), similar to that for RDW (AUC=0.752). Combining

For peer review only - http://bmjopen.bmj.com/site/about/guidelines.xhtml

RDW with APACHE-II score significantly improved prognostic performance (AUC=0.872). Similarly, another study by Wang *et al.*[16] also revealed that adding RDW to APACHE-II score significantly improved prognostic reliability of APACHE-II score in identifying critically ill patients. Therefore, RDW, a simple, inexpensive, and widely available clinical test as part of the complete blood count, may have significant clinical implications for determining prognosis in critically ill obstetric patients. AKI was also significantly associated with in-hospital mortality. However, no significant improvement of prognostic performance was observed when further incorporating AKI into the prediction model.

The pathophysiologic mechanism underlying the association of higher RDW with worse outcomes in critically ill obstetric patients remains unclear. Generally, the increase in RDW reflects either impaired erythropoiesis, abnormal red blood cell survival, or both. Metabolic abnormalities such as shortening of telomere length, poor nutritional status,[30, 31] inflammation, [32-34] oxidative stress, [35, 36] dyslipidemia, hypertension, erythrocyte fragmentation, or alteration of erythropoietin function might contribute to RDW increase.[37] In normal parturition, the increase of RDW might be related to stimulus-induced reticulocytosis in the last few weeks.[30] This might not be the case in our study; wherein, even if five patients with spontaneous onset of labor were excluded, RDW remained as a significant predictor of mortality.

In this present study, the admission rate of 21.73 per 1,000 births was relatively high,[27,38] and the mean APACHE-II score was relatively low compared with other studies.[7, 16, 39] The lack of a high dependency unit as a

For peer review only - http://bmjopen.bmj.com/site/about/guidelines.xhtml

referral center in local areas for complicated pregnancies could partially explain these difference.

This study had several limitations. First, this work did not evaluate potential changes in RDW over time which may provide additional prognostic information. Second, only the APACHE-II score was used to predict mortality in obstetric patients, which was limited by spontaneous improvement after delivery in peripartum women,[40-46] and inconsistent results.[47, 48] Finally, the study was a single-center study. It is warranted to examine the association in different study settings/populations with a larger sample size.

In summary, the study suggested that RDW may be an independent predictor for in-hospital mortality in obstetric patients requiring clinical care. Combining RDW and APACHE-II score significantly improved prognostic assessment among critically ill obstetric patients, which may have direct clinical implications and may aid the improvement of critical care for obstetric patients.

Contributorship statement

Yufeng Chu and Hongsheng Ren conceived of the study, and participated in its design and coordination. Mei Meng, Haiyan Zhou and Chunting Wang extracted data and participated in study design. Yufeng Chu, Zhongshang Yuan and Gong Yang performed the statistical analysis and drafted the manuscript. All authors read and approved the final manuscript.

Competing Interests

For peer review only - http://bmjopen.bmj.com/site/about/guidelines.xhtml

The authors declare that there is no competing interests regarding the publication of this paper.

Fundings

This study was funded by the National Natural Science Foundation of China (NSFC 81200238), the Research Foundation for Excellent Young and Middle-aged Scientists of Shandong Province (BS2011YY043, BS2011YY052),the Young Scholars Program of Shandong University (2016WLJH23), the Clinical medicine science and technology innovation plan of Jinan Bureau of Science & Technology(201602164), the Shandong Provincial Department of Science & Techno-logy research and development plan (2016GSF201052), and the Natural Science Foundation of Shandong Province of China (ZR2013HM062).

Data sharing statement

No additional unpublished data are available.

References

1. World Health Organization. World Health Statistics 2014. Geneva, World Health Organization; 2014.

2. Zeeman GG. Obstetric critical care: A blueprint for improved outcomes. Crit Care Med 2006; 34:S208–S214.

3. Soubra SH, Guntupalli KK. Critical illness in pregnancy: an overview. Crit Care Med 2005;33 (10 suppl):S248-S255.

4. Scarpinato L, Gerber D. Critically ill obstetrical patients: outcome and predictability. Crit Care Med 1995;23(8):1449-1451.

For peer review only

For peer review only - http://bmjopen.bmj.com/site/about/guidelines.xhtml

5. Gopalan PD, Muckart DJ. The critically ill obstetric patient: what's the score? Int J Obstet Anesth 2004;13(3):144-145.

6. Ramsay MM, James D, Steer P, et al, editors. Normal values in pregnancy. London: W.B. Saunders; 2000.

7. Lapinsky SE, Hallett D, Collop N, et al. Evaluation of standard and modified severity of illness scores in the obstetric patient. J Crit Care 2011 Oct;26(5):535.e1-7.

8. Harrison D, Penny J, Yentis S, et al. Case mix, outcome and activity for obstetric admissions to adult, general critical care units: A secondary analysis of the ICNARC Case Mix Programme Database. Crit Care 2005; 9 (Suppl 3):S25–S37.

9. Paternina-Caicedo A, Rojas-Suarez J, Duenas-Castel C, et al. Mortality risk prediction with an updated Acute Physiology and Chronic Health Evaluation II Score in critically ill obstetric patients: A cohort study. J Intensive Care Med 2015 Feb;30(2):97-102.

10. Perkins SL. Examination of blood and bone marrow. In: Greer JP, Foerster J, Lukens JN, Rodgers GM, Paraksevas F, Glader BE, eds. Wintrobe's Clinical Hematology. 11th ed. Salt Lake City, Utah: Lippincott Wilkins & Williams; 2003:5–25.

11. McKenzie SD. Introduction to anemia. In: McKenzie SD, ed. Clinical Laboratory Hematology. Saddle River, NJ: Pearson Prentice-Hall; 2003:161–188.

For peer review only

For peer review only - http://bmjopen.bmj.com/site/about/guidelines.xhtml

12. Tonelli M, Sacks F, Arnold M, et al. For the Cholesterol and Recurrent Events (CARE) Trial InvestigatorsCirculation.Relation Between Red Blood Cell Distribution Width and Cardiovascular Event Rate in People With Coronary Disease. Circulation 2008;117:163–8.

13. Felker GM, Allen LA, Pocock S, et al. CHARM Investigators: Red cell distribution width as a novel prognostic marker in heart failure: data from the CHARM Program and the Duke Databank for Cardiovascular Diseases. J Am Coll Cardiol 2007;50:40-47.

14. Förhécz Z, Gombos T, Borgulya G, et al. Red cell distribution width in heart failure: prediction of clinical events and relationship with markers of ineffective erythropoiesis, inflammation, renal function, and nutritional state. Am Heart J 2009; 158:659–66.

15. Pascual-Figal DA, Bonaque JC, Redondo B, et al. Red blood cell distribution width predicts long-term outcome regardless of anaemia status in acute heart failure patients. Eur J Heart Fail 2009;11:840–6.

16. Wang F, Pan W, Pan S, et al. Red cell distribution width as a novel predictor of mortality in ICU patients. Ann Med 2011 Feb;43(1):40-6.

17. Ani C, Ovbiagele B. Elevated red blood cell distribution width predicts mortality in persons with known stroke. J Neurol Sci 2009;277:103–8.

18. Purtle SW, Moromizato T, McKane CK, et al. The association of red cell distribution width at hospital discharge and out-of-hospital mortality following critical illness.Crit Care Med 2014 Apr;42(4):918-29.

For peer review only

For peer review only - http://bmjopen.bmj.com/site/about/guidelines.xhtml

19. Kim CH, Park JT, Kim EJ, et al. An increase in red blood celldistributionwidth from baseline predicts mortality in patients with severe sepsis or septic shock. Crit Care 2013 Dec 9;17(6):R282.

20. Knaus WA, Draper EA, Wagner DP, et al. APACHE II: a severity of disease classification system. Crit Care Med 1985;13:818–29.

21. Mehta RL, Kellum JA, Shah SV, et al. Acute kidney injury network: report of an initiative to improve outcomes in acute kidney injury. Crit Care 2007;11: R31.

22. Youden WJ: Index for rating diagnostic tests. Cancer 1950;3:32–5.

23. DeLong ER, DeLong DM, Clarke-Pearson DL. Comparing the areas under two or more correlated receiver operating characteristic curves: a nonparametric approach. Biometrics 1988 Sep;44(3):837-45.

24. Sabina Hunziker, Leo A Celi, Joon Lee, et al. Red cell distribution width improves the simplified acute physiology score for risk prediction in unselected critically ill patients. Critical Care 2012,16:R89.

25. Heidi S. Bazick, Domingo Chang, Karthik Mahadevappa, et al. Red cell distribution width and all-cause mortality in critically ill patients. Crit Care Med 2011;39:1913–1921.

26. Weiss G, Goodnough LT. Anemia of chronic disease. N Engl J Med 2005;352:1011–1023.

For peer review only

For peer review only - http://bmjopen.bmj.com/site/about/guidelines.xhtml

27. Hazelgrove JF, Price C, Pappachan VJ, et al. Multicenter study of obstetric admissions to 14 intensive care units in southern England. Crit Care Med 2001 Apr;29(4):770-5.

28. Harrison D, Penny JA, Yentis SM, et al. Case mix, outcome and activity for obstetric admissions to adult, general critical care units: a secondary analysis of the ICNARC case mix programme database. Critical Care 2005;9 (suppl 3):S25-S37.

29. Arabi Y, Goraj R, Horsfall D. Scoring systems in obstetric patients requiring intensive care in Saudi Arabia: a ten year review. Chest 1999;116:286S.

30. Duffy TP. Approach to the patient with anemia. In: Kelley WN, Dupont HL, Glick JH, Harris ED, Hathaway DR, Hazzard WR, Holmes EW, Hudson LD, Humes HD, Paty DW, eds. Kelley's Textbook of Internal Medicine. 3rd ed. Philadelphia, Pa: Lippincott Williams and Wilkins; 1997:1295–1299.

31. Forhecz Z, Gombos T, Borgulya G, et al. Red cell distribution width in heart failure: prediction of clinical events and relationship with markers of ineffective erythropoiesis, inflammation, renal function, and nutritional state. Am Heart J 2009, 158:659-666.

32. Ridker PM, Rifai N, Clearfield M, et al. Measurement of C-reactive protein for the targeting of statin therapy in the primary prevention of acute coronary events. N Engl J Med 2001;344:1959 –1965.

For peer review only - http://bmjopen.bmj.com/site/about/guidelines.xhtml

33. Lippi G, Targher G, Montagnana M, et al. Relation between red blood cell distribution width and inflammatory biomarkers in a large cohort of unselected outpatients. Arch Pathol Lab Med 2009;133:628–632.

34. Sesso HD, Buring JE, Rifai N, et al. C-reactive protein and the risk of developing hypertension. JAMA 2003;290:2945–2951.

35. Kolls JK. Oxidative stress in sepsis: A redox redux. J Clin Invest 2006;116:860–863.

36. Ghaffari S. Oxidative stress in the regulation of normal and neoplastic hematopoiesis. Antioxid Redox Signal 2008;10:1923–1940.

37. Salvagno GL, Sanchis-Gomar F, Picanza A.Lippi G. Red blood cell distribution width: A simple parameter with multiple clinical applications. Crit Rev Clin Lab Sci. 2014 Dec 23:1-20.

38. Rojas-Suarez J, Paternina-Caicedo AJ, Miranda J, et al. Comparison of severity-of-illness scores in critically ill obstetric patients: a 6-year retrospective cohort. Crit Care Med 2014;42:1047–1054.

39. Karnad DR, Lapsia V, Krishnan A, et al. Prognostic factors in obstetric patients admitted to an Indian intensive care unit. Crit Care Med 2004 Jun;32(6):1294-9.

40. Higgins TL, Teres D, Copes W. Updated Mortality Probability Model (MPM-III). Chest 2005;128(4 Suppl):348S.

41. Knaus WA, Draper EA, Wagner DP, et al.APACHE II: A severity of disease classification system. Crit Care Med 1985;13:818–829.

For peer review only

For peer review only - http://bmjopen.bmj.com/site/about/guidelines.xhtml

42. Le Gall JR, Lemeshow S, Saulnier F. A new Simplified Acute Physiology Score (SAPS II) based on a European/North American multicenter study. JAMA 1993;270:2957–2963.

43. Lemeshow S, Teres D, Klar J, et al. Mortality Probability Models (MPM II) based on an international cohort of intensive care unit patients. JAMA 1993;270:2478–2486.

44. Metnitz PG, Moreno RP, Almeida E,et al. SAPS 3 Investigators: SAPS 3–From evaluation of the patient to evaluation of the intensive care unit. Part 1: Objectives, methods and cohort description. Intensive Care Med 2005;31:1336–1344.

45. Moreno RP, Metnitz PG, Almeida E, et al. SAPS 3 Investigators: SAPS 3–From evaluation of the patient to evaluation of the intensive care unit. Part 2: Development of a prognostic model for hospital mortality at ICU admission. Intensive Care Med 2005;31: 1345–1355.

46. Higgins TL, Kramer AA, Nathanson BH, et al. Prospective validation of the intensive care unit admission Mortality Probability Model (MPM0-III). Crit Care Med 2009;37:1619–1623.

47. Stevens TA, Carroll MA, Promecene PA, et al. Utility of acute physiology, age, and chronic health evaluation (APACHE III) score in maternal admissions to the intensive care unit. Am J Obstet Gynecol 2006;194(5):e13-e15.

48. el-Solh AA, Grant BJ. A comparison of severity of illness scoring systems for critically ill obstetric patients. Chest 1996;110(5): 1299-1304.

For peer review only - http://bmjopen.bmj.com/site/about/guidelines.xhtml

For peer review only

Table 1. Baseline clinical and laboratory characteristics by tertile of RDW at critical care initiation

	Tertile I	Tertile II	Tertile III	*P*-value
**RDW (%)**	10.7-13.9	13.9-15.6	15.6-20.4	<0.001
N	131	126	119	-
**Age (years)**	29.6 ± 4.3	29.3 ± 5.6	29.6 ± 5.9	0.84
**Gestational age (weeks)**	35 (32-37)	36 (33-39)	36 (32-38)	0.327
**Primary diagnosis to ICU admission (*n*, %)**				
Hypertensive disorder of pregnancy	50(38.17%)	55(43.65%)	52(43.70%)	0.417
HELLP Syndrome	12(9.16%)	10(7.94%)	8(6.72%)	0.322
Acute fatty liver of pregnancy	10(7.63%)	8(6.35%)	7(5.88%)	0.485
Obstetric sepsis	5(3.82%)	4(3.17%)	3(2.52%)	0.657
Cardiovascular disease	42 (32.06%)	31 (24.60%)	26 (21.85%)	0.162
Gastrointestinal disease	2 (1.53%)	5 (3.97%)	4 (3.36%)	0.476
Stroke	2 (1.53%)	3 (2.38%)	0 (0)	0.332
Pulmonary embolism	1 (0.76%)	0 (0)	1 (0.84%)	0.766
Others	7(5.34%)	10(7.94%)	18(15.13)	0.142
**Hemoglobin (g/L)**	109.5 ± 19.7	100.1 ± 20.7	92.5 ± 24.1	<0.001
**MCV(fL)**	89.8 ± 5.1	86.7 ± 6.0	83.6 ± 11.4	<0.001
**HCT (%)**	32.6 ± 5.8	30.5 ± 6.3	28.8 ± 6.9	<0.001
**APACHE II score (points)**	2 (2-6)	5 (3-6)	10 (6-22)	0.017
**AKI (*n*, %)**	14 (10.69%)	17 (13.49%)	14 (11.76%)	0.207
**TLSH (days)**	8 (7-12)	8 (6-13)	8 (7-12)	0.203
**Hospital Mortality (*n*, %)**	0 (0%)	5 (3.97%)	15 (12.61%)	<0.001

For peer review only

For peer review only - http://bmjopen.bmj.com/site/about/guidelines.xhtml

Note: RDW, red cell distribution width; MCV, mean corpuscular volume; HCT, hematocrit; APACHE-II score, Acute Physiology and Chronic Health Evaluation II score; AKI, acute kidney injury; TLSH, total length of stay in hospital. Values are presented as mean ± standard deviations or number (percentage).

For peer review only

For peer review only - http://bmjopen.bmj.com/site/about/guidelines.xhtml

458 **Table 2. Univariate odds ratios of variables for predicting mortality**

Variables	Odds ratio	95% CI	*P*-value
Age (years)	1.048	0.969-1.133	0.244
Hemoglobin (g/L)	0.997	0.977-1.017	0.763
MCV (fL)	0.962	0.919-1.006	0.098
Hematocrit (%)	0.997	0.934-1.064	0.929
APACHE-II score (points)	1.192	1.124-1.265	< 0.001
AKI (%)	16.61	6.580-42.014	< 0.001
TLSH (days)	0.803	0.691-0.933	0.004
Gestational age (weeks)	1.023	0.920-1.138	0.677
RDW (%)	1.309	1.150-1.489	<0.001

459

460 Note: CI, confidence interval; RDW, red cell distribution width; MCV, mean
461 corpuscular volume; HCT, hematocrit; APACHE-II score, Acute Physiology
462 and Chronic Health Evaluation II score; AKI, acute kidney injury; TLSH, total
463 length of stay in hospital.

464

For peer review only - http://bmjopen.bmj.com/site/about/guidelines.xhtml

Table 3. Independent predictors of mortality by multivariate logistic regression analysis

Variables	Odds ratio	95% CI	*P* value
APACHE-II (points)	1.189	1.071-1.319	0.001
AKI (%)	23.784	6.129-92.296	<0.001
RDW (%)	1.401	1.156-1.697	0.001

Note: Independent variables include age, hemoglobin, MCV, hematocrit, APACHE-II score, AKI, TLSH, gestational age, and RDW. Due to the high correlation between hemoglobin and hematocrit, hemoglobin was first regressed on hematocrit; and placed the residual and hematocrit in the multivariate regression. RDW, red cell distribution width; MCV, mean corpuscular volume; HCT, hematocrit; APACHE-II score, Acute Physiology and Chronic Health Evaluation II score; AKI, acute kidney injury; TLSH, total length of stay in hospital.

For peer review only

For peer review only - http://bmjopen.bmj.com/site/about/guidelines.xhtml

Figure legend:

Figure 1: ROC curve for APACHE II score, RDW, and the combination of both in predicting hospital mortality.

For peer review only

For peer review only - http://bmjopen.bmj.com/site/about/guidelines.xhtml

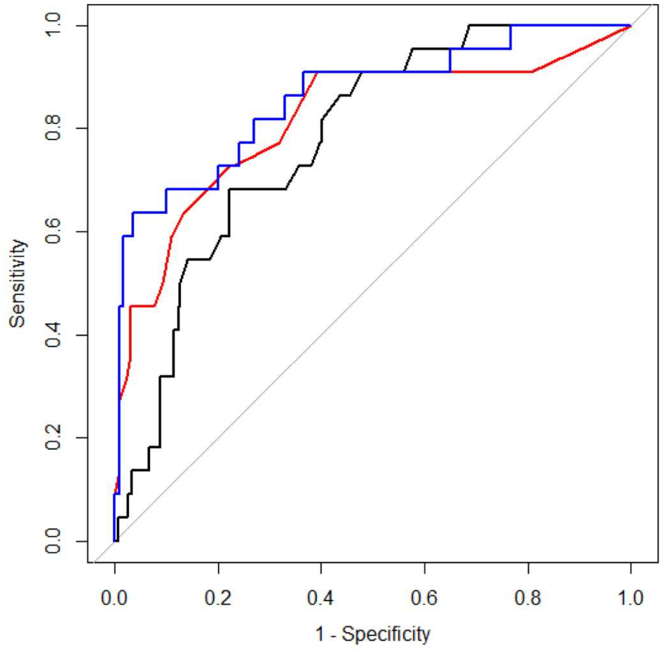

Figure 1

ROC curve for APACHE II score, RDW, and the combination of both in predicting hospital mortality. Compared with the APACHE-II score, adding RDW to APACHE-II scores improved the AUC from 0.766 to 0.872 (*P*<0.001). — RDW (AUC 0.752 ± 0.062); — APACHE-II score (AUC 0.766 ± 0.031); — RDW+ APACHE-II score (AUC 0.872 ± 0.055); ROC, receiver operating characteristic; AUC, area under the curve; APACHE-II score, Acute Physiology and Chronic Health Evaluation II score; RDW, red cell distribution width.

173x177mm (300 x 300 DPI)
